# Selected Instrumental Techniques Applied in Food and Feed: Quality, Safety and Adulteration Analysis

**DOI:** 10.3390/foods10051081

**Published:** 2021-05-13

**Authors:** Graciela Artavia, Carolina Cortés-Herrera, Fabio Granados-Chinchilla

**Affiliations:** 1 Centro Nacional de Ciencia y Tecnología de Alimentos, Sede Rodrigo Facio, Universidad de Costa Rica, San José 11501-2060, Costa Rica; carolina.cortesherrera@ucr.ac.cr; 2 Independent Researcher, Suite 23C, C115A Street, Curridabat, San José 11801, Costa Rica; fabio.granados@ucr.ac.cr

**Keywords:** food and feed research, food chemistry, instrumental food analysis, nutritional quality, safety, authenticity, adulteration

## Abstract

This review presents an overall glance at selected instrumental analytical techniques and methods used in food analysis, focusing on their primary food science research applications. The methods described represent approaches that have already been developed or are currently being implemented in our laboratories. Some techniques are widespread and well known and hence we will focus only in very specific examples, whilst the relatively less common techniques applied in food science are covered in a wider fashion. We made a particular emphasis on the works published on this topic in the last five years. When appropriate, we referred the reader to specialized reports highlighting each technique’s principle and focused on said technologies’ applications in the food analysis field. Each example forwarded will consider the advantages and limitations of the application. Certain study cases will typify that several of the techniques mentioned are used simultaneously to resolve an issue, support novel data, or gather further information from the food sample.

## 1. Introduction

Food is a primary animal and human need; thus, food quality and safety are critical areas of focus in food analysis [1]. As environment and behavior evolve, they will modify food consumption practices [2,3,4]. While consumer trends nowadays gravitate toward healthier [5] and natural [6] food options, an increase in population and high food demand tend to increase the production of processed foods [7,8]. Government and official agencies, then, must concern themselves with enforcing guaranteed food labeling compliance, nutritional quality, and food origin (see, for example, [9,10,11]), as well as the public perception of health and dietary declarations (see, for instance, [12,13,14,15,16] to avoid unfair trade [17].

Food safety is another issue closely related to human and animal health. Forcefully, raw ingredients and foods must be monitored for some of these substances are contaminants of anthropologic origin [18], while others will result from food processing operations or storage [19].

On the other hand, the region of origin or purity of food products and crops (e.g., Protected Designation of Origin and Protected Geographical Indication [20,21]) has demonstrated to be more relevant as consumers search for more specific products (e.g., olives [22], cocoa [23], cheese [24], and wines [25]).

On a related note, food authenticity plays a significant role in food analysis, and some products, such as honey, have a relevant place in the economic pyramid [26]. It is then paramount to assess that the product sold is what the consumer is paying for (e.g., monofloral honey, made up of nectar belonging to a single plant, are more expensive than honey from diverse botanical sources [27]).

Finally, food adulteration can evoke unfair competition and deceit in the final consumer [28], decrease food quality, and even pose a health hazard [29,30]. The consumer and industry demands the research communities to implement assays that might help, in turn, device measures (including regulations) to address the issues mentioned above. This has prompted food scientists to search for new approaches and tools to respond to major current food quality, safety, and authenticity problems.

As an analytical chemistry field investigating analytes using scientific instruments, instrumental analysis has continuously contributed to reaching these goals. As more sophisticated instrumental techniques become increasingly available in food and feed analysis laboratories, food analysis has advanced. It has permitted us to resolve unknowns more efficiently and accurately and explore new food research tools to assess relevant markers (e.g., through food metabolomics [31]).

Herein we will explore analytical techniques as nuclear magnetic resonance (NMR), Polymerase Chain Reaction (PCR), Microwave Plasma Atomic Emission Spectrometer and Inductively Coupled Plasma coupled with Mass Spectrometry (MP-AES and ICP-MS), Isotope Ratio Mass Spectrometry (IRMS), Infrared (IR) and Raman Spectroscopy, OXITEST^®^, as an example of a food accelerated oxidation method, and, finally, Gas and Liquid Chromatography (GC and LC) approaches (especially those using mass spectrometry detection).

An exhaustive explanation of applications of the PCR technique in animal and human feeding was carried out to make known that it is a very valuable technique for this field. PCR applications in food analysis are lesser known techniques that have both quantitative and qualitative capabilities and can supplement other traditional methods. Hence, our intent is to broaden the perspective of its importance for the food industry and for those analysis laboratories that provide services and conduct research on such recent issues that generate controversy in one way or another as in the case of GMOs and allergens.

On the other hand, chromatography techniques have numberless applications and has been highly documented. However, we wanted to expose those emerging analytes in food safety, mainly those that exists usually in low concentrations but can become a health risk such as acrylamide, amines biogenic, and allergens while portraying some of the experience in analyzing them.

**Figure 1:**
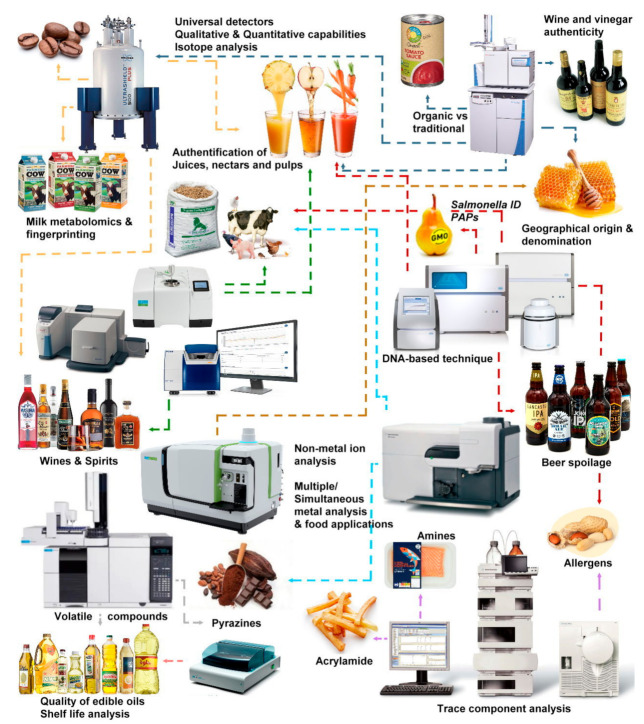
Representation of selected analytical methods and targets to exemplify applications in quality, safety, and authenticity of foods. Each technique complements each other, from each a different amount and nature of information is gathered. Additionally, each technique can benefit from other instrument’s capabilities (e.g., Isotope Ratio Mass Spectrometry (IRMS) benefits from Liquid Chromatography (LC) or Gas Chromatography (GC) separation power).

Hopefully, this review will serve as a primer for scientists to familiarize themselves and comprehend these techniques’ vast capabilities with selected real applications in the food and feed using the most representative and recent examples (Figure 1). The methods below represent approaches that have already been developed or are currently being implemented in our laboratories.

## 2. Selected Instrumental Techniques and Their Applications

### 2.1. Nuclear Magnetic Resonance Spectroscopy (NMR)

Nuclear magnetic resonance bases its principle on the momentary alignment of atomic nuclei (due to an externally applied magnetic pulse) within a sample. More potent instruments have been developed, including a new commercial model by Brüker of 1.2 GHz [32]. An excellent primer for NMR is the recent work by Hatzakis [33]. We also refer the reader to the two reviews regarding NMR in food authentication [34,35]. The constant improvements made in magnet potency has further applications in using this technique (Table 1).

#### 2.1.1. Advantages and Limitations of the Application

As a universal detector, NMR has a definite advantage as multiple analytes/markers can be monitored simultaneously. A myriad of information can be drawn or extracted from a single sample measurement as several nuclei can be used to perform 2D/1D experiments and explore the association among them. NMR also has the advantage of being a relatively fast and versatile, non-destructive, and green approach (as only very small quantities of deuterated solvent are needed to prepare the sample before measurement, and most beverages can be analyzed directly with water suppression pulses). However, it will require a specialized technician that is adept in the NMR sample and instrument manipulation. Additionally, chemometric analysis during food analysis using NMR will be a requirement [61]. Though a less common application, NMR, under some conditions, will allow the user to even use a marker for quantitative analysis (see, for example, [56]). The most relevant disadvantage of NMR-based techniques is that depending on the marker(s) selected for analysis, sensitivity is somewhat limited. Hence, on occasion, researchers will need to complement NMR analysis with other techniques that allow trace analysis (e.g., liquid or gas chromatography). Low-field NMR spectroscopy examples herein (see Table 1) present an opportunity to replace costlier or destructive methods while using non-deuterated solvents [62]. Isotope-based applications using NMR have also been developed and applied to food analysis (see below).

#### 2.1.2. Applications of NMR in Dairy/Milk Metabolomics and Chemical Fingerprinting

To circumvent mass dependence during the assay, Nascimento and coworkers [40] extracted and analyzed the fat fraction of commercial dried milk products. The authors also corroborated their results obtained with NMR by constructing a multivariate model based on NIR. As butyrate occurs in triacylglycerols from milk, this molecule has been used as a marker to determine the composition of fatty acid blends containing milk and non-milk fat ingredients [42]. The authors indicated a high association among NMR data and obtained from a high-resolution GC FAME analysis.

Li and coworkers [38] used metabolite analysis in bovine and caprine milk to determine milk authenticity by discriminating milk to which soymilk has been added. As low as 2 mL/100 mL of soymilk adulteration could be discerned. Palma and coworkers [39] used mammary gland biopsies and milk serum from two different goat breeds (i.e., tolerant/sensitive to feed restrictions). From the analysis of *n* = 50 metabolites, the authors found changes among the control and restricted-fed groups in both breeds, albeit with no differences between breeds. When formulating diets, milk producers should keep in mind these metabolic changes during the scarcity of pastures during the dry season, leading to seasonal weight loss. Recently, Salama and coworkers [48] observed changes in goat milk chemical profiles when animals were subjected to heat stress or treated with *E. coli* lipopolysaccharide (LPS). LPS febrile response was masked in heat-stressed goats. Citrate was increased by heat stress, whereas choline, phosphocholine, *N*-acetylcarbohydrates, lactate, and β-hydroxybutyrate could be considered putative inflammation markers.

Tomassini and coworkers [43] demonstrated that choline (an essential nutrient in human development) and carnitine exhibited differences between two breeds (Holstein and autochthonous Italian breeds). The authors show that 1H-NMR can assess relevant metabolites involved in milk quality both from a nutritional and industrial perspective. Basoglu and coworkers [44] compared the metabolic status of healthy Holstein dairy cows with those suffering from displaced abomasum (a core component of the ruminant stomach that typically resides on the abdomen’s floor fills with gas and rises to the top of the abdomen). The author found the risk of fatty liver and ketosis incremented considerably in cows with displaced abomasum. Also, serum glycine and hippuric acid were found to be reduced in dairy cows with displaced abomasum. Corbu and coworkers [45] performed a metabolomics study on the compositional differences between organic and conventionally produced infant milk. Methionine content was significantly higher in organic-based formulas, which can be used as a convenient marker to differentiate each production system.

Foroutan and coworkers [46] have thoroughly described the chemical composition of Canadian milk samples. Three distinct instrumental analysis techniques were used to evaluate different analytes (i.e., LC-high resolution mass spectrometry [HRMS] with direct flow injection for amino acids, biogenic amines, monosaccharides, acylcarnitines, diglycerides, triglycerides, phosphatidylcholines, lysophosphatidylcholines, and sphingomyelins, ceramides, and cholesteryl esters, LC-MS/MS for the profiling of fat and water-soluble vitamins and free fatty acids, and ICP-MS for trace elements). The authors were able to assign 95% of all visible peaks in the ^1^H-NMR to a specific, fully identifiable compound (for a total of *n* = 59 metabolites in ranges as low as μmol·L^−1^).

Using ^31^P-NMR, García and coworkers [36] compared the phospholipid fingerprint of human, cow, camel, and mare and found species specificity. Camel and mare milk showed higher and lower phospholipid content, respectively. Sphingomyelin and plasmalogens were found in the μg·mL^−1^ range. Boiani and coworkers [37] explored two operation units’ effects during skim milk’s technological processing on the casein micellar structure. In particular, the author examined inorganic phosphate in the serum and the casein-associated phosphate in the retentate. The authors found that a combination of phosphate, calcium, and citrate succeeded in preserve both the casein phosphate nanocluster and micellar casein structure while maintaining retentate pH in the region of the original milk pH. ^31^P-NMR has been used as a routine tool to assess phospholipid content in infant formulas, identifying markers such as phosphatidylethanolamine and sphingomyelin found in the low mg/100 g levels [47].

Finally, water-buffalo milk metabolomics was used to assess differences between conventional and organic farming [41]. Neutral and phospholipid fractions were monitored for two years. A lower milk biosynthesis and lactose levels, phosphatidylcholine, mono-, and polyunsaturated fats characterized milk obtained from conventional feeding systems. Hence, the authors demonstrated that traditional to natural feeding regimes influenced the buffalo milk composition, with possible implications for dairy products’ sensory and nutritional properties.

NMR has achieved considerable advances in metabolomics in dairy research. In this regard, we suggest Sundekilde and coworkers [63] and Maher and Rochfort [64] as primers. Besides, Yanibada and coworkers [65] have prepared a thorough paper regarding the sample preparation methods recommended before metabolomics analysis for bovine milk. Additionally, Bertram [66] highlighted the use of metabolomics in milk and meat to assess authenticity and quality. Recently, Markoska and coworkers [67] have reviewed the most relevant NMR contributions in studies regarding milk protein structural elucidation (especially for β-lactoglobulin, α-lactalbumin, and casein and their fractions/subunits; proteins which have shown resistance to crystallization). Extremely relevant data for protein behavior will determine macro milk behavior during processing. We also suggest the reader an interesting manuscript prepared by Foroutan and coworkers [46], which uses several analytical techniques (including NMR, LC-HRMS, LC-MS/MS, and ICP-MS) and evaluate their contributions to milk composition determination.

#### 2.1.3. Selected Applications for NMR

Recently, Schmitt and coworkers [60] recognized powdered peanuts even from mixtures of similar food products (e.g., almond, hazelnut, walnut) using a marker at 3.05 ppm in the ^1^H NMR spectra. The authors identified the said marker as (2*S*,4*R*)-*N*-methyl-4-9 hydroxy-L-proline after confirmation by synthesis. This approach will serve yet another example of techniques used for allergen detection, a growing health issue.

Hachem and coworkers [58] and Balayssac and coworkers [59] analyzed dietary supplements marketed for weight loss and sold over-the-counter. Despite being labeled as natural products, both groups of authors found contaminated products with non-declared pharmaceutically active ingredients.

Coffee is a standard food product subjected to fraud and then requires authentication. Intuitively, applications for coffee authentication are focused on countries with a trajectory in coffee production. Herein, we included Kenya [56] and Brazil [54,55]. For example, both Ribeiro and coworkers [54] and Milani and coworkers [55] recognized common coffee adulterants in roasted and ground coffee such as barley, coffee husks, corn, rice, soybean, and wheat. On the other hand, Okaru and coworkers [56] focused on quality indicators for coffee and determined caffeine, 16-*O*-methylcafestol, kahweol, furfuryl alcohol, and 5-hydroxymethylfurfural. They also performed fingerprint analysis to evaluate differences among coffee grains from *Coffea arabica* L. and *C. canephora* Pierre ex A. Froehner.

Electrolyte and sugar content makes coconut water a natural isotonic drink, an effective hydrating beverage that is adequate even after a high physical performance [68]. Its pleasant smooth flavor has made coconut water a popular product. Due to its tropical providence and the need for processing due to export demand, coconut water is vulnerable to fraud. Richardson and coworkers [52] used the chemical shift of malic acid as a marker for quantifying the degree of adulteration (i.e., the addition of water and sugars) of Costa Rican coconut water. For an excellent primer regarding the quality, authenticity, and potability criteria for coconut water, we suggest the reader toward a recent review by Burns and coworkers [69].

Monakhova and coworkers [49] used a non-targeted approach to determine if alcoholic beverages were produced illegally or informally and may contain hazardous substances. The authors could form a batch of *n* = 304 samples to determine that *n* = 7 of said samples exhibited divergent NMR profiles. These samples contained undesired contaminants such as diethyl phthalate or polyhexamethyleneguanidine, methanol, or ethyl carbamate. Isaac-Lam [51] used a low-filed NMR to assess ethanol content in wines, beer, and spirits using acetic acid and acetonitrile as internal standards.

Grandizoli and coworkers [50] developed an NMR-based method for quality control assessment of commercial and homemade grape juices by directly analyzing the juice without any sample pre-treatment. The authors followed the acetate and ethanol signals as markers for grape juice quality during refrigeration and room temperature. Recently, Miaw and coworkers [53] explored using a low field NMR to assess grape pulp adulteration with less expensive juices such as cashew and apple juice. The authors were able to identify pure nectars and juices as well as mixtures thereof. The authors compared their results (i.e., analyzed the same samples) using other techniques and demonstrated that NMR constructed prediction models were superior to those performed using ATR-FTIR [70,71,72].

Ogunade and coworkers [57] demonstrated that AFB_1_ altered markers in plasma of lactating Holstein dairy cows (e.g., decreasing alanine, leucine, arginine, acetic acid, and increasing ethanol). Meanwhile, sequestering agents (i.e., sodium bentonite clay and *Saccharomyces cerevisiae* fermentation product) prevented said adverse effects and improved metabolic status. Though it is common to find routine evidence of mycotoxin in the feed [73,74] and the presence of mycotoxigenic fungi (see, for example, [75]), direct evidence of animal exposure to mycotoxins is needed in suspicious cases were veterinary findings were consistent with mycotoxicosis [76].

### 2.2. Real-Time/Quantitative Polymerase Chain Reaction (PCR/qPCR)

PCR is a molecular biological method that involves duplicating and analyzing specific DNA sequences. PCR allows determining whether a particular animal or plant DNA is present in a food product. As such, it can be useful in determining both accidental and intentional adulterations of foods by biological contaminants [77].

#### 2.2.1. Advantages and Limitations of the Application

Though microscopy still the “gold standard” for the detection of Prohibited Animal Proteins or Products (2003/126/EC and 2002/178/EC) [78,79], PCR is a rapid, sensitive, and specific method. Though more expensive (e.g., primer design costs for each animal species must be considered), PCR-based techniques are not dependent on the analyst’s expertise as histological analysis by microscopy [79]. Also, the amount of chlorinated solvent used during the separation of fragments and ingredients during stereoscopic/microscopy analysis is considerable (for some samples, as much as 250 mL per sample). Differentiation of terrestrial mammalian species by microscopy is dependent on the presence of animal hair. In this regard, modern microscopy techniques such as scanning electron microscopy (SEM) permits the identification of the structural markers of such structures with ease (including the creation of new benchtop, economic, SEM models; see, for example, Nikon NeoScope JCM-6000 Plus) [80,81,82,83].

On the other hand, PCR-based techniques require undamaged DNA to be successful. Some of the thermal treatments for feed can render DNA molecules unserviceable. As stated before, improvements in targets and qPCR techniques can already exist even after steaming or autoclaving [84]. Sensitivity-wise, both microscopy and PCR methods can reach similar levels [85]. Finally, Nesic and coworkers [86] compared to light microscopy and PCR methods and applied them to the ruminal fluid of cows fed feed containing MBM as possible forensic significance samples. However, particles of animal origin in the ruminal fluid were detected only by microscopy.

In the case of *Salmonella* analysis, for laboratories that already have an established enrichment-based method, PCR based techniques can be used as a screening technique or a second technique to improve the chances of identifying *Salmonella*-positive feed samples. The PCR-based molecular serotyping method can be done directly with the enriched culture medium and provides a simple and rapid detection and identification for *Salmonella* isolates. Pure culture serotyping for *Salmonella* may typically take 5–10 days for the entire process. Another advantage is that PCR is not dependent on utilizing a substrate or antigens’ expression, thereby circumventing the phenotypic variations in biochemical patterns and lack of detectable antigens [87]. PCR inhibitors and DNA loss during sample processing have already been described during particular feed sample analysis [88,89]. Similar techniques have been developed for the simultaneous detection of multiple pathogens in food for human consumption (see, for example, [90,91]).

In yet another area of application, official methods such as those stated in the GMOMETHODS database [92] are based on the qualitative analysis of modified DNA sequences using PCR and loop-mediated isothermal amplification (LAMP). These methods are the basis for the enforcement of GMO-related legislation for food/feed control as they have been tailored to detect the unauthorized use of GMOs.

As research in allergens grows more robust, more accuracy and sensibility are required from the available analytic and molecular techniques. This is especially relevant to some allergens, as peanut, tree nuts, and milk being the foremost allergens in most countries [93]. However, considerable variation occurs in the individual threshold dose among those with a given food allergy type. For example, from controlled clinical challenge trials in a study of 450 peanut-allergic individuals, peanut thresholds (NOAELs) for peanut-allergic individuals ranged from 0.1 mg up to perhaps 8 g of the whole peanut (an individual peanut can weigh from 500–800 mg) [94]. Westerhout and coworkers [95] recently have derived individual threshold doses from objective symptoms from clinical food challenge data (i.e., based on diagnostic medicine). The work is instrumental as an example of colossal international effort and insights into thresholds (NOAEL-LOAEL). Data obtained through specialized techniques are vital to threshold establishment and will improve the value for precautionary allergen labeling and produce quantitative data to aid in risk assessment [96,97,98].

Several international cohorts and task forces have been assembled in the effort to establish these limits, for example, ILSI Food Allergy—ILSI Europe/iFAAM, EFSA (whose scientific panel has provided several opinions (e.g., [99])), Australia and New Zealand Allergen Bureau’s VITAL^®^, UK Food Standards Agency, FoodDrink Europe, Netherlands Organisation for Applied Scientific Research and the Food Allergy Research and Resource Program at the University of Nebraska-Lincoln to name a few.

DNA-based methods have unparalleled specificity and allow unequivocal identification of most allergens. Additionally, as demonstrated above, multi-allergen screening is not only possible, but it is a trend. In contrast, by its antibody-based nature alone, it cannot be specific. Additionally, enzyme-linked immunosorbent assay (ELISA) has limitations discriminating several allergen targets from other food components (e.g., celery, fish, and almonds). However, PCR is not the preferred method for other allergens: Egg cannot be identified with acceptable sensitivity and specificity, and gluten, a protein, cannot be detected [100]; though methods do exist and coding genes for gliadin (e.g., *GliB1.1* and *GliB1.2*) can be used as targets [101,102]. However, ELISA is a widespread method for detecting and quantitating allergens due to its speed and ease of use, whereas PCR requires a dedicated laboratory space and trained personnel.

Due to the increased number of recognized beer-spoilage species [103,104,105], each species’ evaluation will be excessively laborious and time-consuming. However, multiplex PCR-based techniques permit the simultaneous identification of several known beer-spoilage species. One major drawback of using DNA-based techniques to assess bacteria is the inability to discriminate between viable and non-viable cells. Hence, molecular viability analyses have been developed to circumvent such issues [106]. Notably, in addition to the microorganisms stated above, Enterobacteria (see above) analysis could be used in the beverage industry as the survival of enteric pathogens in beer has been demonstrated [107].

#### 2.2.2. Detection of Animal Products in Feeds (Prohibited Animal Proteins or Products/PAPs)

Most countries inspect their feeds for PAPs as they abide by Commission Regulation (EC) 999/2001, which prohibits the inclusion of protein derived from mammals in feeds for ruminants as PAP produced from ruminant carcasses, is assumed to be the transmission route of Bovine Spongiform Encephalopathy. In this context, molecular assays allow for the automated semi-quantification of nucleic acids, especially when coupled with fluorescent probes.

Fumière and coworkers [108] tested cattle materials treated at 134 °C in various feed matrices down to a limit of detection of about 0.1 g/100 g. The technique was successfully applied to well-characterized meat and bone meal (MBM) samples heated to as high as 141 °C. Similarly, Prado and coworkers [109] exemplified this technique’s application during one of the early interlaboratory studies. Cawthraw and coworkers [110] detected mammalian and avian mitochondrial DNA 16S rRNA genes in animal feed samples. Identification was performed using the 16S rRNA gene from bovine, ovine, porcine, and avian species.

Additionally, vegetable-based feed standards spiked with MBM generated with a commercial rendering process, detecting as low as 0.1 g/100 g MBM. The PCR-based assay was compared with a microscopic examination for a wide range of commercial in several animal feed samples (*n* = 14,678). In this case, *n* = 111 (0.76%) samples examined contained prohibited material. However, most did not represent noncompliance as they were cataloged as pet food. Golinelli and coworkers [84] used qPCR to demonstrate its potential for use in the rapid and routine detection of the presence of bovine, caprine, ovine, porcine, and avian (chicken) MBM in feedstuffs. Kim and Kim [111] developed a multiplex PCR assay using species-specific primer sets and a universal eukaryotic primer for the simultaneous identification set in processed jerky products without DNA extraction. The resulting method showed no cross-reactivity against 16 animal species. It detected as low as 0.1 g/100 g pork in adulterated beef jerky and 0.1 g/100 g chicken in adulterated duck jerky. The meat species of commercial food and feed jerky products and could identify the meat species in commercial jerky products were successful. Finally, in a recent research, Marchetti, and coworkers a high percentage of seafood was mislabeled within the Italian markets [112]. A multi-marker DNA barcoding approach provided reliable and accurate discrimination of shark samples. Using two mitochondrial regions, COI and NADH2, the authors were able to uncover food fraud. This is an essential effort in species conservation and regulating illegal, unreported, and unregulated fishing [112].

Commercial test systems have been already sold as a companion for RT-PCR applications (e.g., r-biopharm SureFood^®^ ANIMAL ID/ANIMAL QUANT tests can be used to prove whether a particular food contains DNA from cattle, chicken, goat, pig, turkey, water buffalo, horse, rabbit, cat, donkey, sheep, and dog). In comparison, there are some commercial tests based on ELISA (e.g., r-biopharm ELISA-TEK™ and MELISA-TEK™).

Similar techniques have also been used to assess meat’s adulteration for human consumption (see, for example, [113,114]). Besides, to this day, some reference materials regarding species identification have been successfully produced to test laboratory adeptness (for example, the Dem Deutschen Referenzbüro für Ringversuche & Referenzmaterialien, which organize proficiency testing schemes for the determination in animal species for food matrices such as gelatin and candy).

#### 2.2.3. *Salmonella* spp. in Feeds

Because some significant outbreaks of human salmonellosis have been traced to contaminated animal feed, rapid and efficient *Salmonella* detection in feed is essential. The determination of *Salmonella* in feeds by immunological or culture-based methods is quite widespread [115]. However, RT-PCR-based techniques to assess *Salmonella* in animal feed became widespread [88,116,117]. In 2014, the European Union organized an interlaboratory comparison study for *Salmonella* in animal feed [118]. Finally, an excellent primer comparing both approaches in *Salmonella* from products of porcine origins can be found in the work by Castagna and coworkers [119]. More recently, Bonilauri and coworkers [120] performed a comparison to detect *Salmonella* spp., *Listeria monocytogenes,* and thermophilic *Campylobacter* spp. in several foods; in all cases, the prevalence was found to be higher using PCR-based techniques.

Soria and coworkers [89] compared two PCR-based methods with several enrichment-based ones. Performance-wise, no method showed significant differences among each other for the identification and isolation of *S. pullorum* and *S. gallinarum* in poultry feed. However, the Salmocyst^®^ broth method showed a 10-fold increase in sensitivity (i.e., as low as 30 CFU/25 g). D’Agostino and coworkers [121] developed a LAMP-based method for the analysis of animal feed for the presence of *Salmonella* spp. LAMP-positive signals are considered presumptive of the presence of *Salmonella*. Simultaneously, confirmation is obtained by continuing ISO 6579, whereas negative reactions can be regarded as signifying that the sample does not contain viable *Salmonella*. With this approach, uncontaminated samples can be identified 24 h less than using the ISO standard method alone; *n* = 13 from the *n* = 79 animal feed samples tested were *Salmonella*-positive. Benahmed and coworkers [122] evaluated the U.S. Food and Drug Administration Bacteriological Analytical Manual lactose broth pre-enrichment medium and peptone buffered water coupled with real-time PCR assays to detect *Salmonella enterica* subsp. *enterica* serovar Cubana in naturally contaminated chick feed. After 24 h of incubation, the qPCR method was as sensitive as the culture method when modified buffered peptone water was used as the pre-enrichment medium but less sensitive than culture when lactose broth was used. After 48 h of incubation, the detection of *Salmonella* Cubana by qPCR and culture in either pre-enrichment medium was equivalent. This indicates that incubation time during pre-enrichment is essential to obtain better results for qPCR. The authors also compared the traditional serotyping method (which uses *Salmonella*’s pure cultures grown on blood agar). Beaubrun and coworkers [123] used the above approach to assess sensitivity in matrixes such as wheat bran, horse feed, dried molasses, calf milk replacer, dried beet pulp, and whole oats. The multiplex PCR serotyping assay applied proved to have application as a rapid screening and serotyping of *Salmonella* contaminating animal feed spiked with as low as 10 CFU/25 g and 2.5 CFU/25 g of *Salmonella enterica* serovars Typhimurium, Agona, and Hadar. Salazar and coworkers [124] analyzed *n* = 21 samples collected from layer hen farms and backyard layers from Ecuador. The researchers used ISO 6579 as a guideline for isolation and PCR to determine strain serotypification. With a high prevalence of contamination (percentages ranging from 33 to 76% from environmental surfaces cloacal swabs, feed, and feces samples), the dominant serovars detected were *S*. Infantis and *S*. Typhimurium. Heymans and coworkers [125] used a multiplex qPCR to assess *S*. Typhimurium and *S*. Enteritidis in several foodstuffs, including animal feed. Pre-enrichment was based on reference method ISO 6579 and a modified version using a Semisolid Rappaport-Vassiliadis medium. Exclusivity for strains tested ranged from 94.6 and 100%, whereas a limit of detection of 40 CFU/25 g was obtained (i.e., similar limit as for the culture-based method). Magossi and coworkers [126] tested 11 feed mills from 8 US states, and 12 sites were sampled within each facility. Samples were investigated both by culture and by PCR analysis for *Salmonella enterica*. From *n* = 237 samples, 66% resulted in culture-positive, and 13.1% were PCR positive, demonstrating the potential role as a vehicle for pathogen transmission and spread into the food production chain. Samar and coworkers [127] also compared traditional techniques and PCR to assess *Salmonella* in vegetables and forages.

Again, commercial test kits have been developed to aid in the fast and semi-automated *Salmonella* assays by RT-PCR (e.g., r-biopharm SureFast^®^ *Salmonella* species/Enteritidis/Typhimurium 4 plex, Bio-rad^®^ iQ-Check *Salmonella* II PCR, Noack *Salmonella* spp. Real-Time PCR, Kylt^®^ *Salmonella* spp.). Recently, PCR techniques have been used to assess multiple foodborne bacterial pathogens [128,129], and other DNA-based methods (nucleic acid sequence-based amplification, LAMP, or DNA microarray) have been reported [130]. Application of PCR in food safety considering other relevant pathogenic Enterobacteriaceae has also been described (see, for example, [131]).

#### 2.2.4. Detection of Genetically Modified Organisms/GMOs

In the work of Fraiture and coworkers [132], the reader can find an excellent primer for GMOs’ detection approaches. On the other hand, Petrillo and coworkers [133] presented DNA target GMO-related sequences by screening public nucleotide sequence databanks by in silico determination of PCR amplification with reference methods for GMO analysis, pooling, and collecting publicly available sequences related to GMOs in food and feed. The JRC GMO-Amplicons database is composed of more than 240,000 amplicons. Barbau-Piednoir [134] used data generated by sequencing and exploited it using BLAST to develop a pragmatic qPCR method to analyze feed additives for the presence of unauthorized a *B. subtilis* (a vitamin B_12_ overproducing GMO). Mano and coworkers [135] validated a GMO detection method based on RT-PCR and increased 10-fold the sensitivity commonly achieved. Commonly introduced target sequences such as 35S [136] and NOS (i.e., *Agrobacterium tumefaciens* nopaline synthase terminator) were selected. Amplification plots obtained demonstrated that the method could discriminate among several GMO-maize events and non-GMO samples (i.e., maize, soybean, canola, and rice), could be applied to processed foods, and with no inhibition. Safei and coworkers [137] recently developed a PCR method for the detection of GM rice. The authors applied the method to *n* = 81 non-labeled rice samples from Iran. The cauliflower mosaic virus (CaMV) 35S promoter and NOS were selected as screening targets for the GM rice sequences. Of the samples tested, *n* = 2 out of 81 (2.4%) samples tested were positive for the CaMV 35S promoter.

Most commercial kits designed to be applied in the laboratory with PCR are based on CaMV-P35S. Some examples include Promega Maxwell^®^ PureFood GMO, Kogenebiotech Powercheck^TM^ (relying on fluorophores like FAM, VIC/HEX, ROX/NED, and Cy5 to improve sensitivity), and Primerdesign^TM^ Genesig^TM^ CaMV 35S Promoter GMO Event quantification kit. Additionally, method ISO 21571 deliver researchers flexibility and can be suitable for an extensive range of matrices [92].

#### 2.2.5. Allergens

Food allergies are an essential health concern worldwide. The presence of undeclared allergenic ingredients or traces of allergens due to accidental contamination during food processing poses a significant health risk to sensitized individuals. Allergic reactions in children can swiftly progress to anaphylaxis, involving respiratory, cardiovascular, or gastrointestinal symptoms [138].

From the regulatory standpoint, Directive 2000/13/EC (which requires manufacturers to declare all ingredients present in pre-packaged foods sold in the EU) has been amended twice concerning allergens (i.e., 2003/89/EC and 2007/68/EC). Other legislative provisions related to food safety include Regulation 1169/2011 and 178/2002. Finally, UNE-EN 15842:2020 and CEN/TR 16338:2012 provide information on the criteria that must be met by molecular biology methods in detecting food allergens. The summary of the selected application of molecular diagnostics for food allergens using PCR is summarized in Table 2.

Therefore, reliable analytical methods are required to detect and identify allergenic ingredients in food products. Real-time PCR allows specific and accurate amplification of allergen sequences. We refer the reader to Linacero and coworkers [145]. The authors review the most recent PCR-based techniques used to determine tree nuts and evaluate the effect of processing and unit operation. Sharma and coworkers [146] have already summarized the most common allergens and techniques (including immunoassay, DNA, and chromatographic-based approaches to assess).

Renčová and coworkers [142] developed a multiplex PCR method to analyze peanut and hazelnut allergens in food matrices. The authors were able to remove inhibition using a 124 bp amplicon (the universal plant primers of chloroplast gene). The resulting method was able to detect as low as 10 mg·kg^−1^ for both nuts. When the technique was applied to the investigation of *n* = 60 commercial food samples, inconsistencies in labeling some foods were found. Xiao and coworkers [139] reported developing an RT-PCR assay to assess the bovine α-lactalbumin gene. The method was able to detect 0.05 ng of bovine DNA and was successfully tested in *n* = 42 commercial food samples with or without cow milk listed as an ingredient. Fernandes and coworkers [147] developed a qPCR method to detect shrimp DNA with low levels as one pg. The RT-PCR method focuses on the 16s rRNA mitochondrial gene. Zhang and coworkers [141] developed a qPCR method to test simultaneously for peanut, soybean, and sesame seed in processed foods. Villa and coworkers [140] used RT-PCR to assess milk proteins in meat-based products detecting as low as 100 and 50 mg·kg^−1^ milk protein concentrate in hams/autoclaved sausages and raw sausage mixtures, respectively. Miyazaki and coworkers [144] detected food allergens, including wheat, buckwheat, and peanuts. Their approach, including reference plasmids containing known copies of the target sequences, could cancel inter-instrument variability and avoid risks of false-positives and false-negatives due to trace levels of contaminants from the laboratory environment or agricultural products. The copy numbers of the plasmids were used to detect the allergenic ingredients to 10 mg·protein·kg^−1^ in highly processed foods. Daems and coworkers developed an alternative to the traditional two-step molecular detection assays (i.e., qPCR followed by a high-resolution melting analysis) applying a fiber optic melting PCR method including mannitol dehydrogenase as a target gene for celery [148].

Interestingly, Suh and coworkers [143] used a multiplex PCR method to assess coding genes from tomato, apple, peach, and kiwi as emerging food allergens. The authors obtained a sensitive method applicable to commercial samples (i.e., juices, dried fruits, and fruit powders). Similar to the other applications mentioned above, commercial kits are available for molecular diagnostics. For example, r-biopharm has a vast catalog of target allergens via qualitative/quantitative PCR (i.e., SureFood^®^ Allergen, using FAM and VIC/HEX fluorescence) that include celery, gluten, soy, almond, Brazil nut, cashew, hazelnut, macadamia, peanut, pecan, pistachio, walnut, crustaceans, fish, mollusks, lupin, mustard, sesame, oat, buckwheat, apricot, and rice.

#### 2.2.6. Beer Spoilage Bacteria

Due to its inherent physicochemical characteristics [i.e., low pH (3.8–4.7), ethanol concentration (0.5–14 g/100 g), and low O_2_ content/high CO_2_ (<0.1 mg·L^−1^ and 0.5 g/100 mL)], beer is quite deterring to bacterial growth, but a few bacterial genera, including *Lactobacillus* spp., *Pediococcus* spp., *Pectinatus* spp., and *Megasphaera* spp. (the first two genera are responsible for 70 to 90% of spoilage incidents, [103]), can generate off-flavors, turbidity, and acidity [149], causing high economic losses and impact brand image. Species-specific PCR tests targeting the gene sequences encoding rRNA have been evaluated to identify breweries’ contamination [150].

Ma and coworkers [151] applied propidium monoazide prior to PCR analysis, allowing live/dead discrimination of bacteria, and used the method to detect beer spoilage bacteria. The intercalating dye covalently binds to the genomic DNA of damaged cell membranes, and once in this state, it cannot be amplified. Schneiderbanger and coworkers [152], during a six-year survey using PCR, demonstrated that the most frequent spoilage-related strains are *L. brevis* (41.9% prevalence), *L. backii* (related to spoilage with early stages of production), and *L. (para-)caseii* (late stages and packaging). Similarly, Meier-Dörnberg and coworkers [153] evaluated the incidence of *S. cerevisiae* var. *diastaticus* beer contamination in Europe throughout ca. 10 years. About six positive contaminations were detected every year, and contamination events caused 71% of them during the beverages’ filling process. Asano and coworkers [154,155] developed a multiplex PCR method to assess *n* = 12 beer-related bacteria’s spoilage capabilities. For practical microbiological quality control in breweries, the authors divided the most common spoilage species into three groups without obtaining false-positive results in any of the multiplex assays.

Similar to other applications, tailored tests have been designed; for example, Rheonix^®^ Beer SpoilerAlert can detect *n* = 60 different organisms to help brewers identify spoilage organisms before spoilage occurs. The assay detects over *n* = 47 different species of lactic acid bacteria, *n* = 7 species of strictly anaerobic bacteria (*Megasphaera* and *Pectinatus*), wild yeast (including *n* = 5 species of *Brettanomyces* and *S. cerevisiae* var. *diastaticus*), and four different hop resistance genes (i.e., *horA*, *horC*, *bsrA*, *bsrB*). Meanwhile, there is the Invisible sentinel^®^ Veriflow^®^ rapid detection, which requires no DNA purification, and Pall GeneDisc^®^ Beer Spoilage Bacteria, a kit that permits the simultaneous detection and identification of *n* = 21 significant beer spoilage microorganisms *L. brevis*, *L. lindneri*, *L. backii*, *L. collinoides*, *L. paracollinoides*, *L. casei*, *L. paracasei*, *L. coryniformis*, *L. rossiae*, *L. parabuchneri*, *L. perolens*, *L. plantarum*, *P. damnosus*, *P. inopinatus*, *P. claussenii*, *P. cerevisiiphillus*, *P. frisingensis*, *P. haikarae*, *P. portalensis*, *M. cerevisiae*, and *M. elsdenii*.

### 2.3. Microwave Plasma-Atomic Emission Spectrometry (MP-AES)

Though microwave plasma technology has been available some decades now, it was not until the introduction of commercially available MP-AES equipment that the technique was popularized [156]. MP-AES uses a 2.45 GHz microwave magnetic field to sustain a nitrogen plasma, and recently, several methods have been developed for the elemental analysis of food samples (see Table 3).

#### 2.3.1. Advantages and Limitations of the Application

To assess the capabilities of metrology, institutes such as the Inorganic Analysis Working Group (IAWG) of Consultative Committee for Amount of Substance: Metrology in Chemistry and Biology (CCQM) from the Bureau International des Poids et Mesures (BIPM) organized the performance test CCQM-K125 (*n* = 25 international participants). The inter-laboratory assay was designed to measure trace elements’ mass fractions (K, Cu, and I) in infant formula. Several analytical techniques to assess these minerals were used, including MP-AES [177].

Overall, MP-AES performance offers better detection limits over a broader range of elements than flame atomic absorption spectroscopy (FAAS) and is usually compared to an ICP-AES [167,173]. The applications above demonstrate a great versatility in matrices that can be analyzed and even sensibilities as low as the ng·mL-1, reflecting the possibility of determining even metals in trace concentrations. Another advantage of MP-AES compared to FAAS is that refractive or carbide-forming elements can be more easily determined because plasma temperature is much higher than those of flame atomizers or graphite furnace [160,161,162]. Despite the virtues concerning FAAS, it still demands the use of only atom lines, additional ionization suppressors (e.g., the addition of CsNO_3_), and the use of internal standards to resolve spectral interferences [156].

A certain advantage of MP-AES is that results are obtained swiftly (analysis of several minerals simultaneously is possible as the MP-AES can screen different wavelengths in a sequential mode) without requiring hazardous (flammable and oxidizing gases) or expensive gases [using nitrogen plasma instead of the argon plasma (flame temperature 5000 vs. 8000–10,000 K)] [168,175]. Hence, spectral interferences are less far-reaching, and atomic spectral lines have superior clarity. The new MP-AES instruments are mostly equipped with nitrogen generators avoiding manual handling of gas-cylinders in the laboratory [158,159,160]. However, torches are highly sensitive to deformation (that can occur even due to wrong placement within the instrument chamber) and tubing wear (used to carry the samples with a peristaltic pump) considerably affects the results obtained MP-AES. For example, the accumulation of salts may cause the torch and nebulizer blockage and may break the torch, so total dissolved salt content present in the samples should also be considered [160,161,162].

As demonstrated through the applications above, the technique can be coupled with a Multimode Sample Introduction System (MSIS) spray chamber for hydride generation that can operate in several modes, which should also be tested for performance for each application [178]. Finally, in other study fields, the combination of MP-AES and chromatographic techniques has also been exploited (see, as examples, [179,180]).

#### 2.3.2. Selected Applications of MP-AES

A work that exemplifies the exploitation of MP-AES’s possibilities was using the technique to analyze *n* = 23 trace elements in sunflower using La, Lu, and Y as internal standards. Concentrations for trace metals As, Co, and Mo were below the MP-AES detection limit. Additionally, the authors compared the results obtained from MP-AES with those obtained in ICP-MS with a high degree of association [156]. Barrientos and coworkers [159] demonstrated MP-AES’s capacity to be coupled with other techniques such as liquid chromatography. The authors developed a SeMet and Se(IV) determination method in yeast, a commonly used supplement in human and animal nutrition. Ozbek and Akman [163] achieved a very sensitive method to assess minerals in commercial bread samples from Turkish markets, including those made with corn, wheat, and rye. Pascariu and coworkers [164] recorded no heavy metal pollution as they analyzed waters from a pristine source to evaluate if MP-AES was sensitive enough to assess low-level contaminants in creek water (from Piule-Iorgovanul Mountains) and if such water was apt for human consumption according to 98/83/EC. Zaldarraiga Heredia [166] compared traditional microwave-assisted digestion with an ultrasound-assisted one to assess multiple elements in Argentine corn, though MP-AES using In as an internal standard. Rajmund and coworkers [167] analyzed *n* = 31 herbal tea samples, an application relevant since fertilization and environmental pollution will influence these plant materials. Calendula tea showed the highest contamination for Cd and Pb (1.65 and 9.12 mg·kg^−1^, respectively). Rodríguez-Solana and coworkers [169] tested *n* = 25 fruit liqueurs from *n* = 16 Portuguese producers. In carob liqueur, 72.7 and 18.2% of samples showed Fe and Pb, respectively. Almond liqueurs contained the lowest mineral content, with only five elements detected; despite sample variability (probably because of the manufacturing processes), a principal component analysis could differentiate among products. Ozbek [168] analyzed *n* = 18 henna samples and demonstrated that several samples exceeded international regulatory limits for Pb. Savoie and coworkers [170] used a cold-vapor technique to assess Hg in wild Atlantic salmon; the results obtained were compared with cold-vapor atomic fluorescence spectrometry without observing differences; levels found ranged from 0.15 to 0.29 mg·kg^−1^ on a dry weight basis. Herman-Lara and coworkers [172] were able to discern, using their mineral profile, among fresh and mature Mexican cheeses and segregate them by geographical origin using the levels for *n* = 9 minerals. Malhat and coworkers [174] applied the technique to determine heavy and essential minerals in honey and analyzed *n* = 100 samples of different botanical origin from Egyptian markets. Mean concentrations of Cd, Cu, Fe, Pb, and Zn were found to be 5, 128, 462, 123, and 244 μg·kg^−1^, respectively. Jung and coworkers [173] compared ICP-OES and MP-AES to assess Mn in Korean wild grape wine. The optimum spectral line (403.076 nm) of MP-AES was different from that (259.373 nm) of ICP-OES, and the authors found a significant matrix effect with the former. The Mn concentration in the wild grape wines was 502–3627 μg·L^−1^.

### 2.4. Inductively Coupled Plasma/ICP-MS for Determination Minerals in Honey

ICP-MS is yet another example of a multi-elemental analysis technique with a vast analytic work range and is capable of hydrides, traces of Hg, and adds capabilities of a semiquantitative, metal speciation, and isotopic measurement. According to Linge [181], ICP-MS has been considered the most recommended technique for determining chemical elements in honey and pollen (see Table 4 for examples). The elemental fingerprint of honey provides essential information with regard to environmental monitoring, authentication, and quality assurance, including nutritional and toxicological aspects.

#### 2.4.1. Advantages and Limitations of the Application

Though ICP-MS is a considerably expensive technique, analyzing several non-metals using spectrometric techniques is far more efficient than other methods (see, for example, [196]). Recently, a green chemistry method using ion chromatography, conductimetric/mass detection, has been developed specifically for honey [197]. ICP-MS is a very attractive technique, especially in high-throughput laboratories. Hyphenated techniques coupled with ICP-MS such as LC can be used to perform multi-elemental speciation [198]. Furthermore, isotope ratios using ICP-MS have been successfully used to assess geographical origin or agricultural products [199]. However, the acquisition or starting price alone can become a hindrance to some laboratories with restricted budgets.

#### 2.4.2. Selected Applications for ICP-MS

Vincevica-Gaile and coworkers [182] analyzed *n* = 80 honey samples from different varietals. In terms of metal concentrations found in the samples, the authors revealed the following sequence Zn > Al > Cu > Ni > Cr > Pb > Co > Cd > As. Similarly, honey types were ranged, in descending order of contamination, as follows: Commercially manufactured honey mixtures with unknown botanical origin > heather/forest blossom honey > multi-floral honey > meadows blossom, honey > linden honey > buckwheat/clover honey > rape/spring blossom honey. Chen and coworkers [183] analyzed *n* = 163 honey samples, including linden, vitex, rape, and acacia, varietals collected from Heilongjiang, Beijing, Hebei, and Shaanxi, China. The authors used principal component analysis, partial least-squares discriminant analysis, and back-propagation artificial neural network to predict sample botanical origin. Conti and coworkers [184] analyzed mostly multi-floral honey from Buenos Aires. The authors used descriptive statistics, hierarchical cluster, and principal component analysis to classify the samples according to their geographical origin. The authors found K and Na to be quantitatively the most abundant metal (i.e., accounting for 92.5% of total minerals analyzed). Mineral analysis of *n* = 19 can also be used as a possible approach to evaluate environmental pollution, though minerals such as As, Be, Cd, Co, Cr, Ni, Pb, Se, Tl, U, and V were found below the limit of detection. Döker and coworkers [182] tested the digestion treatment’s impact (*n* = 4 variants) in the analytical technique sensitivity. Microwave-assisted digestion procedures using diluted reagents emerged as the method of choice for honey. Moreover, *n* = 14 elements were reported for multi-floral honey. The relatively high concentrations of Al, Cr, and V in honey suggested contamination from metallic equipment during processing. Muller and coworkers [185] analyzed three different non-metals (halogens) in honey; their digestion treatment with peroxide produces water in situ that, due to its affinity towards halogens, will hydrate those ions avoiding their loss. Also, the final alkaline pH of digests minimizes memory effects during the ICP-based analysis. Altun and coworkers [189] also determined that honey’s most abundant minerals are K, Na, and Ca. They also used hierarchical clustering to assess the geographical and geochemical precedence of uni and multi-floral honey from *n* = 8 different Turkish provinces. Ataide de Oliveira and coworkers [190] analyzed *n* = 21 different trace minerals in honey and pollen. Pillay and coworkers [191] found several interesting things among the honey samples they analyzed. For example, they determined that Li and Be were metals that were relatively stable across the samples. They also found Cd, Hg, and Pb in 7, 5, 133 μg·L^−1^ each, far below the 1 μg·g^−1^ values permissible daily intake. As honey samples came from *n* = 9, different countries used some mineral ratios (i.e., Cr/Tl, Al/Ni, Bi/Ag) to assess the samples’ origin. Hungerford and coworkers [194] analyzed *n* = 212 samples and found differences among honey from urban, peri-urban, and rural locations. Mineral profiling alone and in conjunction with carbon isotopic ratios have been used to assess honey authenticity [192,195,200]. The above results demonstrate that honey samples’ botanical origin, from mineral profiling, demands a multiplicity of descriptive and inferential statistical and other chemometric analysis [200]. Among the applications mentioned above, some data analysis approaches include linear discriminant analysis, classification and regression trees, artificial neural networking, principal component, and cluster analysis.

### 2.5. Isotope Ratio Mass Spectrometry (IRMS)

Elemental analysis (EA)-IRMS is a bulk measurement technique that provides representative data for the entire sample’s average isotopic signal [201]. High-temperature pyrolysis (ranging up to 1500 °C) has been used as a complement to elemental analysis to assess δ^18^O and δ^2^H as well as performing measurements on δ^13^C in organic samples with a C/O ratio ≈ 1, and δ^15^N in inorganic samples. Consequently, the technique has been for bulk analyses of stable isotopes on light elements [202]. IRMS has also been used in combination with chromatographic techniques [201]. Application for the food analysis method includes identifying natural/artificial sweeteners and food labeling guarantee, assessing the possible substitution of honey with other sugars, quality control of natural juices and carbonated drinks, and determining aroma profile in apple juices, authentication of organic produce. Additionally, isotope ratios of animal tissue from animals can be determined by their feed source, identity the specific feed, and distinguish whether the production was organic or conventional [201]. A smilar approach, regarding production system, has been for fruits such as tomato [203]. For a primer on principles and instrumentation, we refer the reader toward the recent manuscript by Mai and coworkers [204].

#### 2.5.1. Advantages and Limitations of the Application

IRMS can resolve authenticity issues where traditional physicochemical methods are not capable or are limited to assess adulteration (see, for example, Directive 110/2001/EC for honey). Interestingly, for honey (i.e., isotope ratio for honey/protein), fruit juices, maple syrup, wines and spirits, and vinegar the AOAC, the International Vine and Wine Organization (OIV), and the European Committee for Standarization (CEN) has already established δ^13^C and δ^18^O methods to evaluate authenticity (OMA^SM^ 978.17, 998.12, 991.41, 2004.01, TC174 N1048/ENV12140, TC174 N1048/ENV12141, OIV-MA-AS-312-06/OIV OENO 381-2009, OIV-MA-AS2-12/OIV OENO 381-2009, EN 16466-2:2012, EN 16466-3:2012). However, though EA-IRMS can successfully determine adulteration, the hyphenated techniques (e.g., LC-IRMS in honey markers) have demonstrated a vast improvement in sensitivity and robustness [205]. Though extensive research has been accomplished using this technique, work has to focus on harmonizing these emerging approaches [205]. In some cases, techniques complementary to IRMS will be necessary to determine authenticity successfully. An apparent issue with IRMS is that the instrument’s cost is not trivial (more expensive still if a hyphenated technique is desired/required). Maintenance can be cumbersome, as expensive isotopic pure gases will be needed to perform isotope ratio analysis successfully. As demonstrated, simultaneous analysis of several isotopes is possible and suggested, and samples can be determined with minimal or no pretreatment. Initial mass used for IRMS analysis range in the order of μg, hence ensuring sample homogeneity (e.g., sieving; small particle, <1 mm) is vital to obtain accurate results. Finally, other isotope-based techniques, including D/H ratio by SNIF-NMR and ^1^H and ^13^C NMR fingerprinting, have been widely used to build models for the wine varietal and vintage discrimination [206]. In fact, similar to IRMS, official methods using SNIF-NMR have also been established for authentication of fruit juices (OMA^SM^ 995.17), maple syrup (OMA^SM^ 2000.19), vanillin (OMA^SM^ 2006.05), wines and spirits (OIV-MA-AS311-05 and OIV/OENO 381/2009), and vinegar (EN 16466-1:2012).

#### 2.5.2. Geographical Origin of Coffee, and Honey and Coconut Water Authenticity

The stable isotopes composition, such as δ^2^H, δ^13^C, δ^15^N, and δ^18^O, has been used as markers for environmental conditions and agricultural practices, as well as for the identification of the origin of commodities (Table 5, see for example, [207,208]). Honey is a high-value commodity that is prone to adulteration. The majority of plant species use C3 photosynthesis, in which the first carbon compound produced contains three carbon atoms. However, C4 plants (including maize, sugarcane, and sorghum) avoid photorespiration using another PEP enzyme during the first step of carbon fixation [209]. Hence, honey adulteration with high fructose syrups (a product cheaper than pure honey) obtained from C4 plants are easily identified using IRMS, as they reflect their original carbon isotope composition [192]. In this scenario, bees will be more likely to collect nectar and pollen, for their honey production, from C3 flowers and hence will exhibit a different ^13^C/^12^C ratio (or δ^13^C) than those derived from the C4 pathway [210]. An analogous principle is used during coconut water adulteration with sugar [211].

Coffee IRMS-based analysis includes Arana and coworkers [212], who assessed the carbon ratio of coffee from Colombia and compared it to “fingerprints” from other nearby countries. A valuable effort as will help in the efforts to guarantee the country of origin labeling. Additionally, the authors demonstrated that GC-C-IRMS could be an alternative to NMR when the latter is not available. However, as prediction models are based on the number of variables incorporated into a prediction model, NMR is still advantageous as a vast array of molecules can be measured using the said technique. An evident approach to compensate for the stated limitation is the measurement of isotope ratios of several elements (see [214,215], and previous examples within the paper written by Arana and coworkers [212]). Similarly, Barbosa and coworkers [214] created a prediction model of 91.7% accuracy to foretell Brazilian coffee’s origin using combustion and pyrolysis and compared the coffee bean before and after processing (i.e., dehulling/mucilage removal); some relevant differences where observed in the δ^14^N. The authors also contrasted evaluated coffee sensory descriptors vs. altitude.

Although most applications are based on the direct analysis of the untreated samples, Driscoll and coworkers [213] reported a novel approach since they used a spatial model based on δ^18^O after extracting α-cellulose from the roasted coffee samples. The oxygen isotope ratio of cellulose is a useful geographic tracer, as it integrates climate (e.g., humidity, temperature, and precipitation) and source water signals. Similarly, Schipilliti and coworkers [216] used a hyphenated technique to assess isotopic ration in coffee-extracted caffeine. They were able to discriminate based on coffee botanical origin and, in particular, “arabicas” and “robustas”. Peng and coworkers [215] used linear discriminate analysis, *k*-nearest neighbors, and support vector machines to classify the coffees from several Brazil regions. The authors were able to discriminate organic vs. conventionally cultivated coffee using δ^15^N. IRMS technique has also been used in conjunction with other analytical approaches such as mineral profiling (e.g., inductively coupled plasma and X-ray fluorescence spectrometry; [217]).

Wine isotopic analysis for determining the origin and label verification has been used for 30 years [226]. More recently, dos Santos and coworkers [219] used the carbon isotope ratio to confirm that the isotopic signature of the CO_2_ could be derived from C4 sugar’s fermentation. Buzek and coworkers [218] demonstrated that the oxygen isotope ratio from a yet to be described source (i.e., Moravian/Czech Republic wines) could be compared to other European wines for which ratios were previously assessed, highlighting the usefulness of the isotopic ration databank/base construction and curation. Bonello and coworkers [222] used two different analytical techniques and sensory analysis. They distinguished two different sets of red and white wine. They discerned among the two distinct regions (i.e., Veneto and Marches, northwest and central Italy, respectively) from which the grapes used to prepare said wines came from. Fan and coworkers [220] used elemental profiling, based on *n* = 52 elements analyzed by ICP-MS and OES, and oxygen isotope ratio. These variables were incorporated in a multi-step multivariate analysis to verify wines’ origin from three different Chinese regions (i.e., Changji, Changli, and Mile). Perini and coworkers [223] used the δ^13^C of ethanol, Site-Specific Natural Isotope Fractionation, Nuclear Magnetic Resonance (SNIF-NMR), and detected minor sugars using Ion Chromatography with Pulsed Amperometric and Charged Aerosol Detection.

In the case of honey, Zhou and coworkers [192] used stable carbon isotopes analysis in honey and its protein to successfully evaluated questionable authenticity. The authors also incorporated trace elements such as Sr, P, Mn, and K into a predictive model to assess geographic origin. Hence, common and prevalent issues of honey authenticity and the mislabeling of its geographic origin can be identified using a combination of the above two techniques. Kawashima and coworkers [224] the δ^13^C for glucose, fructose, di- and trisaccharides, and organic acids in commercial honey samples employing LC/IRMS and assessed adulterated honey (*n* = 39, 33.6%), demonstrating yet another approach for IRMS.

### 2.6. Vibrational Spectrometric (NIR, MIR, FT-IR (Near-, Mid- and Fourier Transform), ATR (Attenuated Total Reflection), Raman Spectroscopy)

#### 2.6.1. Advantages and Limitations of the Application

Two advantages of these techniques include the price tag of a NIR, MIR, or FTIR system is not as expensive as other techniques listed here. The maintenance required is mostly based on the upkeep of the optic system. The samples require minimal to no treatment, generate no waste or contaminants, determination, and data acquisition is very fast, and the measurement *per se* is highly user-friendly. As in other examples, the strenuous part of the work will be centered on chemometric analysis needed to analyze data and how vibrational bands can be useful for an individual application. Although other techniques can be used as a possible approach to detect adulteration, the less laborious and time-consuming alternative methods, based on vibrational spectroscopy, in the majority of cases associated with chemometric tools, have been developed. Vibrational approaches benefit from being non-destructive and considered green chemistry, while several markers can be selected for predictive model construction.

Other technical approaches to assess juice and pulp adulteration include isotope ratio (SNIF-NMR and IRMS), NMR fingerprinting, and RT-PCR to determine juice authenticity [227]. Furthermore, the mineral profile using ICP-MS has been used to determine the geographical origin and distinguish between conventional and organically grown produce, similar to the applications mentioned herein [227]. In the case of meat authenticity, histological techniques have been developed to determine the disruption of the normal muscle tissue [228,229]. However, this approach requires particular (technically skilled) personnel with specialized knowledge on how to process and interpret histological findings and data and probably even a veterinary and pathology background. Other approaches include DNA fingerprinting, RT-PCR, and its variants, GC, LC/HPLC, ELISA, immunoblotting, and electrophoretic analysis [230]. Each has its own set of limitations, including costs, laboriousness, appropriateness, time-consumption, a diverse range of equipment, and difficulty interpreting obtained results [231]. For an excellent discussion regarding NIR applications in meat processing, we refer the reader to the review written by Dixit and coworkers [232].

As discussed above, research-wise feedstuff analysis using NIR has great potential. NIR applications to assess the quality of feed protein ingredients have been reviewed in the past [233]. However, in terms of feed analysis, for AAFCO Check Sample 2020-28 Cattle Feed, the protein analysis *n* = 118 laboratories used using combustion analysis (AOAC OMA^SM^ method 990.03, AOCS method Ba4e-93) while only *n* = 5 used NIR. Hence, in this scenario, legal decision-making (e.g., label guarantee analysis in official feed laboratories) from data determined by NIR should be restrained at best. Notwithstanding, due to its rapidness, one cannot negate the usefulness of the tool for on-site or in situ analysis and screening. Commercial handheld NIR equipment is available and has been scientifically evaluated for real-time analysis and field monitoring [234,235]. Additionally, good calibration required for quantitative accurate chemical analysis requires is laborious as requires multiple analysis by standard and NIR methods simultaneously. Finally, Raman spectroscopy and hyperspectral imaging have found further food research applications, such as the screening of adulterants in cereals or as a non-destructive approach for the nutritional quality of fruits, determination of antimicrobial drugs, mycotoxins and mycotoxigenic fungi (see, for example, [236,237,238,239,240]).

#### 2.6.2. Detection of Adulterants in Meat and Juices

Several spectroscopy techniques based on molecule vibrational energy have been used to assess adulterants and contaminants [227]. This is especially true for fruit juices included in several listings as the foremost food products at risk of fraud [241]. In meat, authentication problems can be categorized into four major areas where fraud is most likely to occur, i.e., meat origin, meat substitution, meat processing, and non-meat ingredient addition [242]. Thus, adulterations involving bovine, pork, horse, turkey, and llama meats have been detected using different spectroscopic techniques with or without data fusion (e.g., Raman, NIR, MIR; [243,244,245,246]). The most recent examples of vibrational spectroscopy analysis for adulteration of juices, pulps, and meat tissue can be found in Table 6.

As Concord grapes (a cultivar derived from the grape species *Vitis labrusca* L.) have been associated with health benefits, Snyder and coworkers [247] gave themselves to the task to assess the content of Concord grapes in grape juice blends. Nawayon and coworkers [248] prepared *n* = 80 with different sugar additions and could discriminate them using NIR. Alamar and coworkers [249] used NIR analysis as an alternative to analytical methods currently used to evaluate the quality (moisture, total sugars, acidity, soluble solids, pH, and ascorbic acid) of frozen guava and passion fruit pulps. Ellis and coworkers [250] did discriminate diluted juices using FT-IR and the GC-MS analysis of fructose, glucose, and sucrose. Shen and coworkers [251] used both ATR-FTIR and an electronic nose (aromas and volatiles) and GC-MS (flavor profiles) to assess freshly squeezed juice and adulterated juices. A handheld device has also been adapted for in situ monitoring markers of counterfeit alcohol through the container without sample manipulation [253]. Richardson and coworkers [254] used Raman spectroscopy to assess Costa Rican coconut waters’ adulteration by dilution with water and single sugars, mixtures of sugars, and high-fructose corn syrup after pasteurization. Finally, Alamar and coworkers [252] prepared pulps in a pilot plant, and fresh and adulterated samples with sugars and water were assessed using NIR and MIR. It was possible to differentiate adulterated from authentic samples, except for water-adulterated samples using NIR spectra.

Nunes and coworkers [255,256] evaluated non-meat ingredients to increase meat’s water holding capacity in the case of meat tissue. Protein, ash, chloride, sodium, phosphate, and Raman (i.e., 1800 to 700 cm^−1^) and ATR-FTIR spectra were used to determine bovine meat adulteration. Zhang and coworkers [257] used hyperspectral images (i.e., hyperspectral imaging technology can derive a large amount of imaging information at continuous spectral wavelengths and varied spatial dimensions) and near-infrared spectral information of water-adulterated salmon. Yang and coworkers [258] spectrally verified if beef and mutton meat was adulterated with pork meat. A common adulteration practice found commercially since pork meats are considerably less expensive than bovine or lamb meat cuts.

#### 2.6.3. Feedstuff Analysis Using NIR

Aureli and coworkers [259] developed a calibration to predict protein, total P, and phytate-bind P in feed ingredients used for monogastric animal diets. At least *n* = 14 plant ingredients (represented by *n* = 557 samples) were assayed, including cereals and oilseed meals with SEP values of 9.06, 0.80, and 0.66 g·kg^−1^ for each analyte, respectively. Whilst the RMSEP measures the accuracy of prediction, the SEP measures the precision of the projection (i.e., the difference between repeated measurements). Similarly, Fan and coworkers [260] used *n* = 829 samples to assess protein content from several feed ingredients from China’s markets. Ferrerira and coworkers [261] determined dry matter, acid and neutral detergent fiber (ADF and NDF), gross energy, crude fat, ash, and protein to validate a mathematical model then to assess the metabolizable energy of corn (*n* = 99 samples) used in swine feed. Crude ash, fat, and NDF were the variables with the most significant weight. Once the calibration and model are performed, metabolizable energy estimation can be determined swiftly (in a matter of minutes). In contrast, through standard methods, the same assessment would take days or even weeks as it takes considerably longer to get all data from wet chemistry analysis. Metabolizable energy is a useful indicator to assess the animal nutritional requirements assuming losses due to metabolic processes. Alternatively, it will require live animals and a marker (iron oxide is usually used as a fecal marker) to perform metabolism assays. In vitro, dry and organic matter digestibility, and neutral and acid detergent fiber (both indicators useful to assess feed quality) were successfully evaluated by Samadi and coworkers [262]. The digestibility assays’ wet chemistry counterparts usually require two individual 48 h steps for fermentation and enzymatic digestion.

NIR also permits rapid and inexpensive predictions of the nutritional characteristics of forages consumed mainly by ruminants. Karayilanli and coworkers [263] determined the botanical composition of alfalfa and grass mixtures of fresh or ensiled forage; ration consistency and quality based on high-quality forage are paramount to dairy cow productivity. Andueza and coworkers [264] calibrated using *n* = 1040 samples of feces of temperate forage-fed sheep to assess fecal crude ash, protein, fresh forage organic matter digestibility, and voluntary intake. The authors concluded that species-specific calibration models outperform other strategies but entail outstanding maintenance and sample numbers. They also suggest that variability in the predicting model can be broadened using forages from other latitudes, quality, or season. Dry matter determinations for alfalfa, and corn silages obtained with NIR have been compared with those acquired by oven drying and other on-farm methods getting differences as high as 3.5 units [234]. Parrini and coworkers [265] and Yang and coworkers [230] have successfully determined the quality parameters of Italian and Chinese (i.e., *Lolium multiflorum* Lam.) forages. Also, despite broad variability in the plants’ taxonomy and maturity, an Australian research group was able to assess in more than 100 annual and perennial forage species NDF, ADF, pepsin-cellulase dry matter digestibility, and even predicted CH_4_ production during batch culture fermentation [231]. The authors obtained acceptable RPDs (i.e., the ratio of the standard deviation of the reference data is ≥3).

### 2.7. Accelerated Oxidation

Accelerated oxidation tests using OXITEST have now ten years in the market [266]. We selected recent applications using OXITEST^®^ (VELP Scientifica, Usmate Velate, Italy, AOCS Cd 12c-16) as it has demonstrated to be a more accessible, faster, and greener alternative to the RANCIMAT^®^ method (Metrohm, Switzerland, AOCS Cd 12-57) [267]. Most food research using the instrument focuses on the shelf life and antioxidant potential of food products with and without additives (Table 7). Generally, the data obtained from the OXITEST^®^ reactor is expressed as an induction period (IP). Samples are subjected to an environment with high temperature (90 °C) and oxygen pressure (6 atm). Hence, values elevate as the sample is more resistant to oxidation.

#### 2.7.1. Advantages and Limitations of the Application

OXITEST^®^ technology offers rapid information regarding food shelf life that, if not available, would turn the data gathering to be expensive, cumbersome, and somewhat slow; for example, alternatives will require sensory expert panels for rancid perception (see, for instance, [280]. Additionally, The OXITEST^®^ allows measuring the modification of absolute pressure inside the two chambers and, through the OXISoft^®^ Software, automatically generates the IP expressed as hours by the graphical method [280]. Another attractive feature of OXITEST^®^ lies in that accelerated oxidation of whole, untreated samples (i.e., as is or without the previous extraction of fat) can be performed (see, for example, [268]).

#### 2.7.2. Selected Applications for OXITEST^®^

Claus and coworkers [269] combined OXITEST^®^ with hydro- and lipophilic oxygen radical absorbance capacity results and demonstrated that rosemary (*Rosmarinus officinalis* L.) is a promising candidate as a canola oil protectant. Karadag [276] prepared different sunflower oil emulsions in water-mediated by sunflower lecithin, whey protein isolate, whey protein concentrate, citrus pectin, Tween 80, and the mixture of Tween 80 and Span 20 at two different pH (i.e., 4 and 7). The author demonstrated that lecithin and pectin showed the highest and lowest induction periods’ values, respectively, at both pH values. These results are relevant since many food formulations can be classified as emulsions, and is in this interface that oxidation occurs.

Morina and coworkers [271] demonstrated that fresh calf meat (i.e., three hours after slaughter) showed a high IP while prepared meat (possibly due to manipulation) exhibited the lowest values. Meanwhile, blended meats and sausage containing peppers and onions revealed the overall highest IP values. Riciputi and Caboni [270] compared the Italian bakery product’s IP when prepared using different cooking oils. They demonstrated that IP was improved when the preparation was made using extra virgin olive oil instead of sunflower oil. They also showed that coarse milling of ingredients increased significantly the IP, a particularly relevant result that can be considered during baked goods formulation to improve shelf life. However, coarse grinding can have a negative effect on palatability. Marzocchi and Carboni [272] synthesized tyrosyl oleate and incorporated it into the formulation for tarallini, which improved the IP four-fold compared to the control sample (from 6.10 to 25.28 h using 7 g/100 g oleate). The authors also monitored the baked goods’ volatile profile that resulted from the Maillard reaction, caramelization, and lipid oxidation using an SPME-GC/MS method. Shan and coworkers [273] demonstrated that steaming pork has a detrimental impact on IP values compared to raw meat. Simultaneously, the addition of 40 g/100 g pickled dried mustard increased this value ca. two-fold, without affecting the sensory quality of the meat. Thanomwongwatana [274] added a polyphenol-rich extract from *Tamarindus indica* L. seed husks to feed. Extracts at 0.5 and 1.0 mL/100 g had the highest antioxidation potentials, with the average IP readings at 5.42 and 5.43 h, respectively. Grape skin extract in concentrations of 5 000 mg kg^−1^ was also used to improve walnut paste’s IP values [275]. Oleynikov [277] demonstrated that beef prepared with oregano extract exhibited higher IP values than beef formulated with additives E262, E300, E301, E331, and E391, which means that oregano extract was better equipped to slow down myoglobin oxidation in meat. Similarly, Romeo and coworkers [278] demonstrated that polyphenols from olive mill wastewaters used to enrich sunflower oil improved its resistance considerably to oxidation by 50% (IP of 1 022 min) with respect to the control IP of about 540 min. Several examples demonstrate the use of natural antioxidants to improve food properties, but they establish a vast and relatively untapped potential to exploit agro by-products.

Amato and coworkers [268] compared the nutritional quality of chia seeds produced from Italy’s southern region from those commercially available IP values for the whole seeds. Peruvian seed exhibited a higher IP than mineral and organically grown Italian and Australian seeds. Also, HPLC-MS was used to identify *n* = 34 metabolites from leaves tentatively. Zhang and coworkers [279] determined the fatty acid composition of *Paeonia ludlowii* (Stern & G. Taylor) D.Y. Hong to assess its potential as food. GC/MS was used to determine fatty acid and volatile profile (where oleic and α-linolenic acids and cinnamene, and 1,3-xylene were among the most abundant compounds, respectively). The authors also demonstrated that tea polyphenols’ addition to the crude oil improved its IP (i.e., 7.41 h shelf life at 25 °C of 200.73 days) considerably.

### 2.8. Gas Chromatography, Coupled with Mass Spectrometry Detection (GC/MS)

Gas chromatography has a myriad of applications in food quality and safety [281], is an emerging technique, which has several advantages (including swiftness), compared to LC. Nevertheless, GC requires volatile, semivolatile, or thermally stable analytes or labor-intensive chemical derivatization techniques for separation [281]. A GC equipped with an MS detector is an even more powerful tool for food analysis. A crucial forte of this technique is its high-resolution separation and broad usage by the food industry for analyzing target compounds, metabolomics (targeted and untargeted), and volatile compound profiling [281].

In terms of food safety, GC/MS is the preferred method to assess persistent organic pollutants (compounds that accumulate in the environment and organisms). Known examples are dioxins and polychlorinated biphenyls (PCBs) (i.e., toxic undesired by-products of industrial processes and waste incineration) [282,283]. Yet another example of residues usually assessed by using both GC and LC are pesticides thata are usually extracted via fast approaches such as QuEChERS [284]. Pesticides analysis by GC/MS is so common that is even included in undergraduate curricula [285]. Both techniques are also routinely used in the assessment of food contamination via packaging materials (e.g., phtalates). For this point we refer the reader to an excellent primer by Bernaldo de Quirós and coworkers [286].

#### 2.8.1. Advantages and Limitations of the Application

One of the main advantages of using GC coupled with MS spectrometry includes the capability of screen simultaneously total ion chromatograms and selected ion monitoring, which will give a broader perspective of all compounds present in a mixture. In contrast, ion monitoring will achieve sufficient S/N ratios to obtain very high sensibilities. Additionally, GC databases are widespread, continuously expanding, and are valuable to identify unknown compounds [287,288]. The main drawback using GC/MS is that all analytes must be able to be volatilized and then be thermally stable. Additionally, some carrier gases can be expensive (He among the most used gases; ca. 500 USD for 220 scf). For volatile compounds, ion mobility spectrometry has been used to assess quality during chocolate manufacture [289].

#### 2.8.2. Organic Species of Mercury in Water and Marine Biota Tissue

Mercury (Hg) and its compounds are of much concern for their high toxicity, bioaccumulation, and their widespread presence in the environment. There is enough evidence that fish and marine life are considerably affected by mercury species, and this contaminant can be directly introduced to the human diet [290,291]. Since the toxicity of Hg is species-dependent, various methods have been developed for the speciation analysis of Hg in several matrices [292,293]. GC is a convenient alternative for organic mercury species as they are volatile (Table 8). The determination of organic mercury species is especially relevant as there is evidence to associate them with total mercury contents in human blood [294].

Watanabe and coworkers [295] co-injected polyethyleneglycol to suppress adsorption of methyl phenyl mercury (a common trait in the measurement in GC/MS is using sodium tetraphenylborate). The authors also used acetone and toluene to reduce the possibility of emulsion formation. Lipids and sulfur-containing amino acids have been described as matrix interferences that have been dealt with using copper ions and freeze-drying during extraction [296]. Applying headspace and a polydimethylsiloxane (PDMS) fiber as a vehicle during solid-phase microextraction (SPME) resulted in a highly sensitive water analysis method. Such an approach could be practical for other matrices. Interestingly, normalized water analysis methods are usually based on GC/MS (see, for example, ISO 21863).

Other hyphenated techniques such as HPLC-ICP-MS have been used successfully to analyze organic mercury species (see, for example, [298,299]). However, GC/MS’s availability is far more abundant than these techniques as the latter system configuration is less expensive. Other methods for arsenic, selenium, and mercury speciation have already been described previously [300]. Additionally, Nevado and coworkers [301] compared different gas chromatography-based hyphenated techniques (e.g., GC-atomic fluorescence spectrometry; Carrasco and Vassileva [302] used this technique to assess MeHg in marine biota).

#### 2.8.3. Volatile Compounds/Pyrazines (Py) in Cocoa

Flavor is one of the most crucial quality properties of cacao beans, playing a pivotal role in cocoa products’ admissibility, such as cocoa powder. Alkalization and roasting were two critical steps in cacao beans processing that can affect the final cocoa powder flavor. Besides, pyrazines are flavor compounds formed during the roasting stage by the Maillard reaction (Table 9).

Among the *n* = 5 geographically different liqueurs tested, Liu and coworkers [303] highlighted that Papa New Guinea liqueur exhibited a better profile both employing gas chromatography-olfactory-mass spectrometry (i.e., higher content of volatiles including tetramethylpyrazine, tetraMePy) and sensory (i.e., preferred by the panel) based analyses. da Veiga Moreira and coworkers [304] analyzed the volatile compounds of fermented cocoa beans and chocolate produced from different hybrids cultivated in Brazil. The authors demonstrated that aroma and protein (as per matrix-assisted laser desorption/ionization time of flight [MALDI-TOF] MS) profiling could be used as a crop genetic improvement parameter. Alasti and coworkers [305] performed extensive research that focused on the effect of processing in cocoa powder production (i.e., *n* = 5 steps were monitored for volatile compound content: Raw ingredients, alkalinization, roasting, milling, pressing, and the product). Among the compounds identified, 2,3,5,6-tetramethylpyrazine increased almost tenfold from the cocoa beans to the powder. The authors also demonstrated that alkalization and roasting were the foremost steps in the cacao beans processing that can affect the final cocoa powder flavor. Clark and coworkers [306] revealed that artisan processing (i.e., chocolate refining, melanging) that the type of melanging influenced the overall aroma profile (based on *n* = 88 compounds), and time had a more significant effect than the temperature in the production of dark chocolate. Hamdan and coworkers [307] introduced encapsulated, and nano emulsified *Spirulina platensis* carotenoids into dark and milk chocolate and demonstrated that untreated chocolate contained overall fewer pyrazines content.

### 2.9. Liquid Chromatography with Mass Spectrometry Detection (LC/MS^n^)

LC/MS has become necessary in the chemical analysis of foods. The technique has been used to identify and quantify bioactive compounds such as fat-soluble and water-soluble vitamins [308] and in the determination of carotenoid and polyphenol profiles for the characterization of some foods of interest due to possible biological activity [309,310].

Liquid chromatography is powerful tool in the field of food safety [311], being fully implemented in both routine analysis and research laboratories. LC-MS is considered essential for the monitoring of food contamination. Of most concern for health are naturally occurring toxins and environmental pollutants.

Naturally occurring toxins include mycotoxins [312,313], marine biotoxins [314,315], cyanogenic glycosides [316], and toxins from poisonous mushrooms (e.g., γ-amanitin, ustalic acid) [317,318]. In the latter example, toxins are usually measured mostly in biological samples such as plasma, serum, and urine as measure of exposure [319,320].

Additionally, antimicrobials, especially those with agronomical applications, are routinely scrutinized in medicated and non-medicated feed (as they are used for prophylaxis, methaphylaxis, and growth promotion) [313,321,322], in animal tissue [323] and other essential commodities (e.g., eggs, vegetables) [312,324,325,326]. LC-MS has also a widespread application in the determination of pesticide residues in foods, as the accumulation of these substances may cause toxic and allergic effects for health as a result of the consumption of contaminated products [327,328].

In the following subsections, we focused our review mostly in analytes that are generated by food processing or storage such as acrylamide and biogenic amines. Furthermore, this technique is even used to analyze more complex molecules such as peptides from allergy proteins. Finally, we included an example using LC-MS to detect fraud (i.e., vanillin).

#### 2.9.1. Advantages and Limitations of the Application

Among its multiple advantages, LC-MS can be applied to various analytes, from those with low molecular weight to more complex molecules such as peptides. It is possible to simultaneously determine several analytes of the same nature, such as several allergens peptides of whole egg, skimmed milk, and soy flour ground hazelnut, and ground peanut in a cookie [329,330]. Even LC-MS could be used to analyze analytes of different natures, as in the case of simultaneous determination of acrylamide and hydroxymethylfurfural in extruded products [331]. Among the limitations is the high-cost equipment, and the data analysis is complex and requires time. As mass detectors are very sensitive, inert polymers (e.g., PTFE) must be used during sample preparation and high purity solvents (LiChrosolv^®^ MTBE price is ca. 160 US for 2.5 L) and stable isotope labeled standards (acrylamide-*d*_3_ price is ca. 100 USD for 5 mL) can be also quite expensive. Finally, some chromatography separations may require solvents considered to be environmentally problematic (e.g., CH_2_Cl_2_, MTBE) or solvent waste may be considerable.

#### 2.9.2. Acrylamide

Acrylamide has been classified as a possible carcinogen by various organizations around the world [332]. Acrylamide is commonly found as a Maillard reaction product (i.e., foods containing free amino acids, mainly asparagine, and reducing sugars subjected to intense heat treatment, usual temperatures above 120 °C, such as frying, roasting, or baking) [333].

According to the FAO-WHO report “Health implications of acrylamide in food” in 2002 [334], acrylamide was found in almost all the foods analyzed, including cookies, cereals, bread, coffee, with fried foods being these type of product with the highest content.

Research on acrylamide content in certain foods, their manufacturing conditions, and habits of consumption have become relevant in recent years as per these contaminant risk assessment [335].

Since 2002, several publications have been developed around analytical methods to determine acrylamide in food and techniques including liquid chromatography, gas chromatography, capillary electrophoresis, and immunological tests, being the first two with the most significant number of applications in food [336,337,338].

Acrylamide is a compound with low molecular weight, high polarity, and significant solubility in water. The molecule has a simple chemical structure without chromophores, so the use of spectrophotometric techniques is limited (i.e., UV or fluorescence detectors). Also, the quantification of acrylamide in complex matrices, such as foods, is hindered by various compounds that act as interferences. Hence, MS detection is a useful tool for determining this analyte sensitively and selectively.

One of the main aspects to consider when working with LC-MS or GC-MS techniques is the sample preparation; most publications mention that a homogenization process is necessary because acrylamide levels in the food are low (mg·kg^−1^ or μg·kg^−1^). The analyte could be extracted with aqueous solutions or polar solvents such as methanol [331,339], but in samples with a high-fat content (potato chips, roasted nuts, cocoa beans, olives, nut and peanut paste, desserts, prepared meals), it is necessary to defat them with hexane [335,340,341].

Salt solutions such as Carrez (I and II) are frequently used to eliminate some interferences such as protein material or starches in coffee, nuts, snacks, deserts [342], but it useful to treat matrixes such as potatoes chips [343,344,345], bread samples [346], and olives [347]. Recently some researchers [335,341,348] used a dispersive extraction method using QuEChERS (i.e., mostly mixtures of MgSO_4_ and NaCl) to perform extraction and purification. Under this approach, the components’ proportions can be modified according to the matrix fat and protein content [349,350,351].

The purification and concentration of the acrylamide can either be attained by solid-phase extraction (SPE), from a hydro/lipophilic balance stationary phase (e.g., OASIS^®^ HLB) or strong cation exchange (e.g., OASIS^®^ MCX) [346,352].

From the instrumental standpoint, most authors consider that the use of an internal standard (i.e., acrylamide-*d_3_*) is paramount to compensate analyte loss along with the extraction (i.e., recovery) and measurement (i.e., ionization) steps [339,341,346,352].

Regarding the chromatographic conditions used in liquid chromatography, most of the publications describe a reverse-phase approach (i.e., C_18_ columns and mixtures of acidified MeOH, ACN, and water as mobile phases; either acetic or formic acid, to ensure ionization of the acrylamide molecule). Most researchers used electrospray ionization in positive mode, and multiple reaction monitoring (MRM) of transition ions, in general, is m/z 72.0 → 55.0 and 75.1 → 58.0 m/z for acrylamide and the internal standard, respectively. Acrylamide detection using LC-MS provides exceptionally high sensitivity and accuracy, with reported detection limits as low as 2 μg·kg^−1^ and recoveries usually above 85% [337,341,342].

#### 2.9.3. Biogenic Amines

Other compounds that are important as markers in food quality and safety are biogenic amines (BAs). As acrylamide, BAs are products from different reactions suffered by certain free amino acids generated by proteolysis processes during some foods’ storage due to some microorganisms’ (MOs) intrinsic metabolism. These MOs can be part of the associated flora of food or introduced by contamination before, during, or after processing [353,354,355,356,357,358].

BAs are produced by amino acid decarboxylation (i.e., reaction catalyzed by decarboxylases) and aldehyde and ketone transamination (i.e., reaction catalyzed by transaminases). The BAs are low molecular weight, non-volatile and thermostable compounds; they are classified according to their structure into aliphatic amines such as putrescine, cadaverine, and agmatine, aromatic amines such as tyramine and phenylethylamine, or heterocyclic amines such as histamine and polyamines such as spermidine and the spermine [354].

Biogenic amines could be found in fresh foods such as vegetables, fruits, meats, or fish since they are matrices with high protein content and can undergo proteolysis during storage [359,360,361,362,363,364,365]. The content in fresh products is not high enough to cause maximum intoxication. There are intestinal amine oxidases that can rapidly metabolize and detoxify these compounds. In particular, biogenic amines are found in a higher concentration in those foods that are obtained by a microbial fermentation process, such as cheese, beer, wine, soy-based ferments, and fermented dry sausages [355,356,360,361,362,363,364,365,366].

Biogenic vasoactive amines, such as histamine, tyramine, and β-phenylethylamine, are responsible for immediate and short-lived responses in the inflammation process, including vasodilation, increased vascular permeability, and smooth muscle contraction. Histamine toxicity is also known as “scombroid poisoning,” caused by eating spoiled fish of the Scombridae and Scomberesocidae families (tuna, mackerel, bonito, bluefish, and so on). Tyramine intoxication, known as the “cheese reaction,” is associated with ripened cheese consumption. However, high levels of this amine have also been observed in meat and meat products [353,358].

Symptoms that occur from biogenic amine poisoning include nausea, shortness of breath, hot flashes, sweating, heart palpitations, headache, bright red rash, burning mouth, and hypo or hypertension. Most of them are generated by histamine and tyramine. In the case of amines such as putrescine, cadaverine, agmatine, and spermidine, they can react with nitrites present in food, mainly in meats and sausages, and generate nitrosamines, compounds that have been classified as potentially carcinogenic [356].

International organizations as US FDA and European Commission have established some concentration limits for biogenic amines in food, especially histamine. For example, 50 mg·kg^−1^ and 100 mg·kg^−1^ in fish [364]. Different countries have established diverse limits of amines for wine (e.g., 2, 5–6, and 8 mg·L^−1^ for Germany, Belgium, and France, respectively) [365]. In yet another beverage, beer, the Slovak Republic has set their limit for histamine at 20 mg·kg^−1^ [366]. The recommended limits for 2-phenylethylamine and tyramine are 30 mg·kg^−1^ and 100–800 mg·kg^−1^ food, respectively [367].

Some studies have established biogenic amine indices (BIA) that describe, in some way, the quality of various foods (meat, fish, wines, etc.) and indicate the freshness or deterioration degree. Histamine, tyramine, cadaverine individually or a combination of several amines such as putrescine-cadaverine, spermidine-spermine are used for this BIA. Miet and Karmas [368] used a BIA to observe the decomposition of fish, which is based on the increase in the levels of putrescine, cadaverine, and histamine and the decrease in the levels of spermidine and spermine during the storage process, as BAI = (histamine + putrescine + cadaverine)/(1 + spermidine + spermine). Scores of 0 and 1 indicate good quality fish, between 1 and 10 are tolerable, and a score of more than 10 indicates spoilage of the product. Hernández-Jover and co-workers [369] also established BAI for freshness in the meat where <5 mg·kg^−1^ indicating good quality fresh meat, between 5 and 20 mg·kg^−1^ for acceptable meat but with signs of initial spoilage, between 20 and 50 mg·kg^−1^ for low-quality meat, and >50 mg·kg^−1^ for spoiled meat. In general, ratios < 0.5 correspond to good products, whereas values > 0.7 are associated with an advanced state of decomposition [357].

There are a plethora of publications regarding analytical methods for the determination and quantification of biogenic amines, including spectrophotometry, fluorimetry, electrophoresis, immunoassays [including ELISA], biosensors, and chromatography [353,354,355,356,357,358]. The LC technique is the most used for food analysis. However, biogenic amines have weak chromophores in their structure, so many of these methods use UV, DAD/PDA, or FLP detectors [359,362,368] and, hence, derivatization techniques (pre-column or post-column). The pre-column derivatization is more sensitive and is, therefore, used more frequently than post-column derivatization [353,354,358,359,360]. Recently, Munir and Badri [358] described in detail derivative compounds to use derivatizing agents, though the most commonly used are dansyl chloride and *o*-phtaldehyde.

It should be noted that if the derivatization process is required, it should be carried out as recommended by Sentellas and coworkers [357]. We also recommend the work by Salazar and Castro [370]. They developed an experimental design to evaluate which are the determining factors of the process: Temperature, time, the volume of the derivatizing agent, among others.

Though derivatization-based methodologies are widespread, they are tedious, usually lengthy, as reactions need to be controlled, and are highly susceptible to interferences such as lipids or proteins present in the matrix, which could cross-react with the reagent and decrease the sensitivity of the technique [358,359,360,371]. Biogenic amine analyses have been recently carried out using mass detectors, which brings several advantages such as a shorter sample treatment and greater sensitivity [356,358] but with results comparable [364] to the methods mentioned above.

As with other techniques, LC-MS requires a thorough sample homogenization and extraction of amines, where pH care is vital since it is necessary to know the state of the analyte (neutral or ionized); most extraction methods involve acid solutions [356,371]. Positively charged amines are easier to extract and detect. As per clean up, the purification of extracts can be performed using SPE [362,364] or matrix solid-phase dispersion [363,364]. The compound 1,7-diaminoheptane has been a valuable molecule to use as an internal standard [365]. 

The separation and quantification conditions usually reported include reversed C_18_ phases [364,371,372], mobile phases as ammonia buffers, ACN, and MeOH [365,371,372]. Such conditions can be applied to detect several analytes simultaneously. For example, Sagratini and coworkers successfully separated eight biogenic amines in fish [371]. Regarding detection, most researchers use electrospray ionization with positive polarity (ESI^+^) [369] due to the cationic nature of amines and multiple reaction monitoring (MRM) [355,372]. The mass spectrum’s base peak and isolated precursor ion was an m/z signal that corresponds to the protonated molecule [M+H]^+^ [371,372]. 

#### 2.9.4. Allergens

It is estimated that worldwide there is 4% of the adult population (i.e., 18^+^ years of age) that suffers from some food allergy, while the results from 2009–2010 show that in boys and girls, the incidence of allergies due to food consumption is 8% [373]. In fact, the last 20 years have seen an increase in reported cases of food allergies. Hence, most of the International regulatory framework focuses on the prevention of cross-contamination within food industries, correct and precise labeling of food about the presence (or not) of allergens, and a clear understanding of the effect of processing on allergens.

To achieve the aforementioned objectives, it is necessary to have precise and robust analytical methodologies. ELISA kits are widely known to be the most common and available on the market; it has a detection rate of 0.1–5.0 mg·kg^−1^, but their sensitivity may be compromised by thermal processing and generate false positives. ELISA kits are limited as each kit can only be used for a unique type of allergen, so the analysis of several allergens implies a high cost of analysis and time [330,374]. After that, the use of LC-MS for the detection and quantification of allergens in food has become more advantageous since it is possible to perform simultaneous analysis of several allergens in a single matrix with high sensitivity, selectivity, and time reduction [330,374,375,376] (see additional examples in Table 10).

As with other applications, sample preparation is pivotal to a successful analysis. In the case of allergens, what is being analyzed are proteins, to be more specific peptides. Hence, the sample is through enzymatic digestion with trypsin, which is selective in the cleavage of the C-terminal proteins to lysine and arginine and generates peptides whose lengths generally fall within a range susceptible to analysis by MS [330,376,377]. As in any extraction process, it is necessary to optimize parameters, like the composition of the extraction buffers, temperature, sample–buffer ratio, and the presence of detergents [383,388]. The purification and concentration of the extracts are also paramount, where SPE is mostly applied [330,374] (Table 10).

LC separates the extracted peptides based on differences in affinity relative to a stationary phase, usually C_18_ [374] and a mobile phase corresponding to acidifed solutions and acetonitrile. Later, the eluting peptides are then ionized depending on the type of equipment; these can be the tandem MS (also known as MS/MS or MS^2^), ESI-MS, MALDI, and surface-enhanced laser desorption/ionization (SELDI-TOF-MS) [375,376,377] (Table 10).

For the detection of allergens, selecting marker peptides is necessary, usually performed first on raw ingredients before analyzing processed foods. It is possible to carry out this selection instrumentally using HRMS and computational algorithms such as MASCOT [389], X! Tandem, SEQUEST. Another selection tool is “in silico peptide selection,” in which computational software and protein databases are used to perform a simulated digestion [388].

The selected reaction monitoring (SRM), also known as multiple reaction monitoring (MRM), is used to quantify allergens based on both precursor ion and production [329,330,383,389,390]. It is the most critical part of the analysis, and it requires time. It is also primordial using stable isotope analogs of labeled peptides (SIL) to compensate for the influence of matrix effects on ionization [376,383,387,391]. Notwithstanding, SIL is not suitable for analyzing different effects that arise during sample preparation [330].

#### 2.9.5. Vanillin

Natural flavoring ingredients such as vanilla extracts are of widespread usage in the food and beverage industry. However, other artificially obtained compounds (e.g., ethyl vanillin, coumarin) may be added to reduce costs. LC-MS is a practical mean to differentiate among artificial/natural vanilla extracts (Table 11).

Interestingly, coumarin as a food ingredient is even regulated in some matrixes under regulation EC 1334/2008 (2020 act). In this regard, we suggest and excellent primer by Lončar and cowokers [395].

Additionally, various metabolomic approaches have been developed for vanilla profiling. For example, Gu and coworkers [396] performed a comparative metabolomics analysis by LC-MS and compared vanilla profiles before and after curing to determine vanillin biosynthesis. The authors found that at least seven different putative pathways of vanillin biosynthesis, which may correlate with microbial activity. Busconi and coworkers [397], based on 260 phenolic compounds (including flavonoids, lignans, stilbenes, and other polyphenols), were able to distinguish among vanilla originated from Papa New Guinea as “Tahitian vanilla” (i.e., *Vanilla × tahitensis* traditionally cultivated on the islands of French Polynesia).

Despite most applications are based in liquid chromatography, another research group has used GC equipped with vacuum ultraviolet spectroscopy to assess these compounds [398]. The authors were able to assess guaiacol, veratrol, piperonal, eugenol, 4-hydroxybenzaldehyde, vanillin, ethyl vanillin, coumarin, vanillic acid, caffeine, piperonal, eugenol, and 4–hydroxybenzaldehyde as markers for authentication. Finally, Lamprecht and coworkers used both IRMS δC^13^ and HPLC and found that they do discriminate the authenticity of vanilla extracts. In IRMS authentic Madagsacar pods δ_PDB_^l3^C = −18.7 to −21.5, while values more negative than −21.5 were therefore considered to be adulterated [399]. Similarly, GC-IRMS δ^13^C and pyrolysis-based δ^2^H were used to distinguish among vanilla varietals and trace them to their geographical origin [400]. More recently, MID-FTIR was used to predict values of ethylvainillin and coumarin in pure vanilla extracts, while corroborating their results with LC [401].

## 3. Conclusions

The selected applications of the above techniques in food analysis demonstrate that answering complex questions and practical problems will require more than one technique or even several techniques. In this scenario, some of these techniques can become redundant (give equivalent information to the researcher) or complement each other. Additionally, some of the applications demonstrated herein are multivariate, for which they are dependent on the ability of a user to handle and interpret complex statistical descriptive and inferential data analysis (in addition to the specialized knowledge needed to operate highly technical scientific equipment). The researchers must evaluate before acquiring or even before attempting to solve any scientific questions using any or some of these techniques, their limitations, and advantages to assess whether the approach is in line with the scientific inquiry to be answered. Researchers must also carefully consider the availability in their laboratory and the costs to routinely upkeep some of these expensive instruments. In this scenario, multi- and inter-disciplinary teamwork among agencies, Universities, laboratories, and even specialized technicians, scientists, and personnel are advantageous. The data compiled here have demonstrated the versatility, range, and pertinence of several analytical techniques whose specific applications within the food analysis context can help solve relevant questions and research needs that usually arise within the industry and academia.

## Figures and Tables

**Table 1:** Examples for NMR spectroscopy for food analysis.

Matrix	Application	Method, Solvent	Nucleus	Frequency, MHz	Reference
Milk					
Mammalian milk	Phospholipid fingerprinting	^1^H decoupling	^31^P	400 (161)	[36]
Skim milk	Ionic strength effect on micellar casein during membrane separation and diafiltration	1D, D_2_O	^31^P	500 (202)	[37]
UHT Milk	Authenticity	1D and 2D, HSQC, and HMBC, D_2_O	^1^H, ^13^C	400 (100)	[38]
Mammary gland secretory tissue and milk serum	Two goat breeds	1D NOESY	^1^H	800	[39]
Milk powder	Fat content	Time-domain transverse relaxation	^1^H	9	[40]
Water Buffalo milk	Conventional vs. biological feeding	1D, D_2_O, Carr–Purcell–Meiboom–Gill) pulse sequence	^1^H, ^13^C, ^31^P	400 (100, 161)	[41]
Milk	Butyrate marker unknown fat blends	1D, broadband, and inverse gate decoupling	^13^C	400 (100)	[42]
Milk	Holstein Friesian vs. autochthonous Italian cows	1D and 2D, DOSY, TOCSY, HSQC, HMBC	^1^H, ^13^C	400 (100)	[43]
Serum, urine, and liver	Displaced abomasum in Holstein cows	1D NOESY, D_2_O	^1^H	600	[44]
Formula milk	Organic vs. conventional production	1D NOESY	^1^H	500	[45]
Commercial bovine milk	Chemical composition analysis	1D, *T_1_* NOESY, D_2_O	^1^H	700	[46]
Infant formula	Phospholipid fingerprinting	1D, ^1^H decoupling	^31^P	400 (161)	[47]
Goat milk	Mastitis and heat stress	1D, D_2_O	^1^H	600	[48]
Beverages					
Alcoholic beverages	Hazardous substances (diethyl phthalate or polyhexamethyleneguanidine, MeOH, ethyl carbamate)	1D, D_2_O, 1.5 mol·L^−1^ KH_2_PO_4_ pH 7.4	^1^H	400	[49]
Grape juice	Authenticity	1D, D_2_O, zgpr	^1^H	400	[50]
Wine, beers, spirits	Alcohol content	1D, one pulse	^1^H	45/400	[51]
Coconut water	Water-sugar mixtures	1D, zgesp,	^1^H	800	[52]
Grape pulp	Authenticity (adulteration with apple or cashew juice)	1D, Carr-Purcell-Meiboom-Gill pulse sequence	^1^H	15	[53]
Processed coffee seeds					
Roasted and ground coffee	Authenticity	1D, zg30 and ZGCPPR	^1^H	600	[54]
Roasted and ground coffee	Authenticity	1D, zg30, D_2_O		600	[55]
Commercial coffee samples	Quality and authenticity	1D, zg30, qNMR	^1^H	400	[56]
Toxins/Biological samples					
Bovine blood	Aflatoxin ingestion biomarker	NOESY, D_2_O, and 250 mmol L^−1^ KH_2_PO_4_, pH 7.0	^1^H	700	[57]
Dietary ingredients					
Food supplements	Adulteration with phenolphthalein, sildenafil, fluoxetine, lorcaserin, orlistat, and sibutramine	Qualitative, CD_3_CN:D_2_O; qNMR: MeOD	^1^H	500	[58]
Food supplements	Adulteration with phenolphthalein and sibutramine	MeOD	^1^H	59.7	[59]
Allergens					
Foods	Adulteration with peanut, (2*S*,4*R*)-*N*-methyl-4-9 hydroxy-L-proline (marker)	NOESY, CD_3_Cl, MeOD	^1^H	400/600	[60]

**Table 2:** Selected method details for applications of PCR in allergens.

Organism/Target Gene	Primer Sequence (5′-3′)	Amplicon Size, bp	Reference
Single allergen analysis			
*Bos taurus* α-lactalbumin	*α-LA*-F: CACCCAGGCTGAACAGTTAACA *α-LA*-R: TCCGTAGCCCTTCAAGTCTTTC Probe: FAM-AGGTGTTCCGGGAGC-MGB	67	[139]
*Bos domesticus* 12S rRNA gene	916-F: GTACTACTAGCAACAGCTTA 916-R: AGACTGTATTAGCAAGAATTGGTG Probe: FAM-TCTAGAAGGATATAAAGCACCGCCAAGT-BHQ1 EUK-F: AGCCTGCGGCTTAATTTGAC EUK-R: CAACTAAGAACGGCCATGCA Probe: FAM-AGGATTGACAGATTGAG-BHQ2 18SRG-F: CTGCCCTATCAACTTTCGATGGTA 18SRG-R: TTGGATGTGGTAGCCGTTTCTCA	121, 120, 113	[140]
Multiplex allergen analysis			
*soy28k* (soybean/*Glycine max (L.)* *Merr.*), *2S albumin* (sesame/*Sesamum indicum* L.), and *Ara h 1* (peanut/*Arachis hypogaea* L.)	*Soy28k*-F: CTAGAAACATTGGAAACACC *Soy28k*-R: ATCACATACCCTCAAGACAT *Ses i 1*-F: TGAGGAACGTGGACGAGAG *Ses i 1*-R: CCCTAGCCCTCTGGTAAACC *Ara h 1*-F: CCATCATTTCACCATCCACAC *Ara h 1*-R: CTCTCATTGCTCCTGCTACTA 18S rRNA-F: TCTGCCCTATCAACTTTCGATGGTA 18S rRNA-R: AATTTGCGCGCCTGCTGCCTTCCTT	147, 126, and 82	[141]
Peanut (*Ara h 1*) and hazelnut/*Corylus avellana* L. (*Cor a 1*)	*Ara h 1*-F: AGAGGGAGATATCACCAACCCAATC *Ara h 1*-R: GAGTTGAAGTGTGGGAGCATCAAAG *Cor a 1*-F: AAAGGCCATCAAGAGCATTG *Cor a 1*-R: CATCGCCTTCAATCACACTG *Chloroplast*-F: CGGACGAGAATAAAGATAGAGT *Chloroplast*-R: TTTTGGGGATAGAGGGACTTG	180, 258, 124	[142]
Tomato (*Cyclophilin*), Apple (*Mdtl 1*), Peach (*Pru p 2.01A*), and Kiwi (*Pectin methylesterase inhibitor*).	Pru-F: GCAACCGGAATTAGCAAC Pru-R: AAATCTTGACCCCCGTTCTC 18S rRNA-F: CGAAAGCATTTGCCAAGGAT 18S rRNA-R: CCGGAACCCAAAGACTTTGA Sola 5-F: GGAGCCAAATTCAACGATG Sola 5-R: ACGACGTGCTTTCCGTTGA Act 6-F: AAATCTGTCCCAAAACTCGC Act 6-R: TTAGCACTGGCCTGAGCTAT Mal 2-F: CTTGCCTTGCGTTTGGTGAT Mal 2-R: GGCACTGCTTCTCAAAGATCTCA	209, 172, 146, 127, and 105	[143]
Wheat, buckwheat, and peanut	F: CAT GGT GGG CGT CCTC R: AAA GGC CAT AAT GCC AGC TG Probe: FAM-CGG ATG CAC TGC ITT GAT AAA G-MGB F: CGT TGC CGA GAG TCG TTC TGT TT R: CGC CAA GGA CCA CGA ACA GAA G Probe: FAM-CGG GAC GCG CTT C-MGB F: TTG GTT CAA AGA GAC GGG CTC R: CAC GAG GGT TGT TCT CGA CC Probe: FAM-ACC GCG GCA GAT GG-MGB	64, 101, and 71	[144]

**Table 3:** Selected applications in food matrices and parameters for Microwave Plasma-Atomic Emission Spectrometry (MP-AES) analysis.

Matrix	Sample Treatment	Minerals Tested and Wavelengths Used (nm)	Sensibility, mg·L^−1^ or mg·kg^−1^	Reference
Sunflower *^a^*	Microwave digestion HNO_3_	Al 394.401/396.152, As 193.695/234.984, Ba 455.403/614.171, Be 234.861, Ca 393.366, 422.673, Cd 226.502/228.802, Co 340.512/345.351, Cr 357.868/425.433, Cu 324.754/327.395, Fe 259.940/ 371.993, K 766.491/769.897, La 394.910, Lu 261.542, Mg 285.213/383.829, Mn 403.076/403.307, Mo 379.825/386.410, Na 588.995/589.592, Ni 341.476/352.454, Pb 368.346/405.781, Sr 407.771/ 460.733 88, Y 371.029, V 309.311/437.923, Zn 213.857/481.053	0.20 × 10^−4^	[156]
Animal Feed	Microwave digestion HNO_3_	Cu 324.754, Fe 259.940, Mn 257.610, and Zn 213.857	1.5 (Mn) to 4.1 (Fe)	[157]
Aquaculture feed	Dry ash	Cu, Fe, Mn, Zn, K, and Na	0.4 to 3.9	[158]
Biofortified yeast *^b^*	Methanesulfonic acid digestion 16 h 120 °C, heptafluorobutyric acid and K_2_S_2_O_8_ + NaOH/HCl/NaBH_4_ + NaOH (hydride formation)	SeMet and Se 196.026	(3.80 and 7.60) × 10^−4^	[159]
Malbec wines *^c^*	Dilution HNO_3_ and ethanol	Sr 407.771, Rb 780.027, Mg 279.553, Ca 396.847, Na 589.592, K 769.897	1.0 × 10^−3^	[160]
Bread *^a^*	Wet digestion HNO_3_/H_2_O_2_	Ca 393.366, Cu 324.754, Fe 371.993, K 766.491, Mg, 285.213, Mn 403.076, P 214.915, Zn 213.857	2.8 × 10^−4^ (Cu) to 7.5 (P)	[161]
Cheese *^a^* (several varieties)	Wet digestion, HNO_3_ + H_2_O_2_, Cs as a suppressor	Ca 445.478, K 766.491, Mg 285.213	0.012 (Mg) to 0.19 (Ca)	[162]
Wines *^a^*	Standard addition, dilution ethanol	B 249.772	0.08	[163]
Natural water	Filtrate	Al 396.152, Cd 228.802, Co 340.512, Cr 425.433, Cu 324.754, Fe 259.940, Mn 403.076, Mg 285.213, Mo 379.825, Ni 352.454, Pb 405.781, and Zn 213.857.	0.005	[164]
Californian wines *^b^*	Dilution HCl/KI (reduction)	As 193.695	3.8 × 10^−4^	[165]
Corn *^a^*	Microwave digestion HNO_3_	Ag 328.068, Al 396.152, Ba 455.403, Be 234.861, Ca 422.673, Cd 228.802, Co 340.512, Cr 324.433, Cu 324.754, Fe 259.94, Mg 383.829, Mn 403.076, Mo 386.41, Na 589.592, Ni 352.454, Pb 283.305, Tl 535.046, V 437.923, Zn 481.053	0.7 (Mo and Fe) to 4.3 (Ca)	[166]
Drinking water *^b^*	None, NaBH_4_/HCl	As 188.979, Se 196.026/203.985, and Hg	0.007 (Hg) to 0.04 (As)	
Herbal tea infusions	Filtrate	Cr, Mn, Fe, Ni, Cu, Zn, Cd, Al, Pb, Co	0.01 to 0.12	[167]
Henna *^a^*	Microwave digestion (HNO_3_/H_2_O_2_)	Al 396.15 B 249.77, Cd 228.80, Co 340.51, Cr 425.43 Cu 324.75 Fe 371.99, Mn 403.08, Mo 379.83, Ni 352.45, Pb 405.78 Sn 317.51	0.30 (Cu) to 8.43 (Ni)	[168]
Carob, fig, and almond liquors	Wet (HNO_3_/H_2_O_2_/HCl/HClO_4_) and dry ash mineralization (450 °C/HNO_3_)	Na 588.995, K 766.491, Cu 324.754, Ca 393.366, Mg 403.076, Na 588.995, K 766.491, Fe 371.993, Zn 213.857, Mn 403.076, Cd 226.502, Pb 368.346 and P 213.618	0.05 (Cu) to 0.52 (Ca)	[169]
Salmon *^b^*	Microwave digestion H_2_O_2_ + HNO_3_/NaBH_4_ Thiourea	Hg 253.652	0.02	[170]
Water *^a^*	Tetrahexylammonium bromide/4-(2-pyridylazo)-resorcinol/ ethanol/HCl	Pb 283.305, Cd 228.802, Co 345.351, Ni 305.082, Zn 213.857, and Cu 324.754	0.06 (Cu) to 4.9 (Cd)	[171]
Goat cheese	Dry ash (550 °C 5 h and HCl/HNO_3_)	Pb 405.781, As 193.695, Cd 228.802, Al 396.152, Ca 393.366, Mg 285.213, K 766.491, 588.995, Co 340.512, Cu 324.754, Cr 425.433, Fe 371.993, Mn 403.076, Se 196.026, Zn 213.857, Ni 352.454, Sr 407.771	0.023 (Mn) to 100 (Na)	[172]
Wine *^a^*	Wet digestion H_2_O_2_/HNO_3_	Mn 403.076	0.67 × 10^−4^	[173]
Honey *^a^*	Dry ash (450 °C for 4 h) and HNO_3_/H_2_O_2_	Cu 324.75, Fe 259.94, Pb 405.78, Zn 213.86, and Cd 228.80	0.29 to 4.5	[174]
Coffee, tea, cocoa	Dry ash 600 °C for 4 h and HNO_3_	Mn, Cr, Fe, Cu, Zn, Pb, and Cd	<0.1	[175]
Related matrices				
Blood *^b^*	HNO_3_ + H_2_O_2_/KI/NaBH_4_ + NaOH (hydride formation)	Sb 231.147	1.50 × 10^−4^	[176]

*^a^* Double-pass glass cyclonic spray chamber. *^b^* MSIS spray chamber*. ^c^* O-ring free glass baffled cyclonic spray chamber.

**Table 4:** Minerals analyzed and applications of ICP-MS in honey.

Location of Origin	Sample Treatment	Instrument used/Minerals Tested or Isotopes Used	Concentrations Found	Sensibility	Reference
			μg·L^−1^ or μg·kg^−1^		
Latvia	Wet digestion, dilution/HNO_3_ + H_2_O_2_	Al, As, Ba, Cd, Ce, Co, Cr, Ni, Pb, Rb, Sr, V	1.15 × 10^3^ (Al) to 1.14 × 10^1^ (Pb)	<2	[182]
China	Microwave digestion, HNO_3_ + H_2_O_2_	Agilent 7700x, ^23^Na, ^24^Mg, ^31^P, ^39^K, ^43^Ca, ^55^Mn, ^56^Fe, ^63^Cu, ^66^Zn, ^85^Rb, ^88^Sr, and ^137^Ba	5.52 × 10^5^ (K) to 9.00 × 10^1^ (Cu)	290 (K) to 0.2 (Mn)	[183]
Argentina	Microwave digestion HNO_3_	Dynamic reaction cell Elan, Perkin Elmer, As, Be, Ca, Cd, Co, Cr, Cu, Fe, K, Mg, Mn, Na, Ni, Pb, Se, Tl, U, V, Zn	8.16 × 10^5^ (K) to 3.00 × 10^1^ (Cr)	<10	[184]
Turkey	Microwave digestion HNO_3_ and HNO_3_ + H_2_O_2_	Agilent 7700x, ^24^Mg, ^27^Al, ^44^Ca, ^55^Mn, ^56^Fe, ^63^Cu, ^66^Zn, ^85^Rb, ^88^Sr, and ^7^Li, ^51^V, ^52^Cr, ^59^Co, ^60^Ni, ^69^Ga, ^75^As, ^78^Se, ^101^Ru, ^105^Pd, ^111^Cd, ^121^Sb, ^125^Te, ^133^Cs, ^137^Ba, ^178^Hf, ^193^Ir, ^195^Pt, ^205^Tl, ^208^Pb.	1.20 × 10^2^ (Cu) to 1.25 × 10^4^ (Mg) and 0.6 (Tl) to 42.1 (Cr)	<1 (Mn, Cu, Zn, Rb, Sr) to 100 (Ca) and 0.2 (Tl, Sb) to 217 (Li)	[185]
Romania	Microwave digestion HNO_3_ + H_2_O_2_	Agilent 7500, Ag, Al, As, Ba, Be, Ca, Cd, Co, Cr, Cs, Cu, Fe, Ga, K, Li, Mg, Mn, Na, Ni, Pb, Rb, Se, Sr, Tl, U, V and Zn	2.29 × 10^5^ (K) to 1 (Tl)	0.25 (Rb) to 118.3 (K)	[186]
Brazil	Microwave digestion, cellulose, NH_4_NO_3_, NH_4_(CO_3_)_2_, NH_4_OH	^79^Br, ^127^I	5.81 × 10^2^ to 3.84 × 10^3^ (Br), 9.9 (I)	34 (Br), 6.0 (I)	[187]
Brazil	H_2_O_2_ + NH_4_OH/single reaction chamber system (UltraWave^TM^)	ELAN^TM^ DRC II, Perkin Elmer-SCIEX, ^79^Br, Cl, and ^127^I	2.60 × 10^2^ to 1.51 × 10^3^ (Br), 7.00 × 10^4^ to 3.21 × 10^5^ (Cl), 42 (I)	<30 (Br) 1.00 × 10^4^ (Cl), and 5 (I)	[188]
Turkey	Microwave digestion HNO_3_ + H_2_O_2_	Agilent 7500 ce, ^25^Na, ^27^Al, ^39^K, ^44^Ca, ^52^Cr, ^55^Mn, ^56^Fe, ^60^Ni, ^63^Cu, ^66^Zn, ^78^Se, ^111^Cd, and ^208^Pb	4.55 × 10^4^ (K) to 4.56 × 10^1^ (Mn)	<1	[189]
Brazil	Microwave digestion HNO_3_ + H_2_O_2_	Agilent 7700x, ^45^Sc, ^75^As, ^79^Se, ^89^Y, ^111^Cd, ^115^In, ^139^La, ^140^Ce, ^141^Pr, ^144^Nd, ^150^Sm, ^152^Eu, ^157^Gd, ^162^Dy, ^165^Ho, ^167^Er, ^169^Tm, ^173^Yb, ^175^Lu, ^208^Pb, and ^209^Bi	13.84 (As) to 0.04 (Yb)	1.20 × 10^−4^ (Tm) to 3.1 (Pb)	[190]
UAE	Microwave digestion, 3 mL/100 mL HNO_3_	Li, Be, Ag, Cd, Sb, Hg, Tl, Bi, Th, U and Pb, Se, V, Ni, Cr, Al	1 to 25 and 1.00 × 10^2^ to 4.00 × 10^3^	<1	[191]
Australia and International	Wet digestion HNO_3_ 100 °C, 2 h	Agilent 7900, Ag, Al, As, Au, B, Ba, Be, Bi, Ca, Cd, Ce, Cs, Cr, Co, Cu, Dy, Er, Eu, Fe, Ga, Gd, Ge, Hg, Hf, Ho, Rb, K, La, Li, Lu, Mg, Mn, Mo, Na, Nb, Nd, Ni, Os, P, Pb, Pd, Pt, Pr, Re, Ru, Se, Sb, Sr, Sm, Sn, Ta, Tb, Te, Th, Tl, Tm, Ti, U, V, W, Y, Yb, Zn, and Zr	1.00 × 10^6^ (Na) to 1.00 × 10^2^ (Sr)	Non indicated	[192]
Serbia	Microwave digestion HNO_3_ + H_2_O_2_	As, Cu, Zn, Fe, Cd, and Pb	3 (Cd, As) to 2.21 × 10^3^ (Fe)	1 (As, Cd) to 120 (Zn)	[193]
Australia	Microwave digestion, HNO_3_	Agilent 8800 Triple quad, Ag, Al, As, B, Ba, Ca, Cd, Co, Cr, Cu, Fe, Hg, K, Mg, Mn, Mo, Na, Ni, P, Pb, Sb, Se, Sn, Sr, V, and Zn	2.5 (Hg) to 9.65 × 10^5^ (K)	5 (As, Hg, Pb) to 5.00 × 10^3^ (P)	[194]
Romania	Microwave digestion HNO_3_ + H_2_O_2_	Al, As, Ba, Ca, Cd, Co, Cr, Cu, Mg, Mn, Na, Ni, K, Pb, Sr, Tl, V, and Zn	1.90 × 10^5^ (K) to 5 (As)	0.50 (Sr, Pb) to 5.00 × 10^3^ (K)	[195]

**Table 5:** Selected applications for IRMS (coffee and wine geographical origin analysis and the determination of honey and coconut water authenticity).

Technique	Sample Presentation	Number of Samples	Countries	Isotopes Measured	Reference
Coffee					
GC-C-IRMS	Roasted coffee	34	Colombia, Brazil, Peru	δ^13^C	[212]
IRMS (on α-cellulose)	Roasted coffee	49	Brazil, Burundi, Colombia, Costa Rica, El Salvador, Ethiopia, Guatemala, Honduras, India, Indonesia, Kenya, Mexico, Nicaragua, Panama, Papua New Guinea, Peru, Rwanda, Tanzania, USA, Vietnam, Yemen	δ^18^O	[213]
EA-P and EA-C-IRMS	Green beans	24	Brazil	δ^13^C, δ^18^O, δ^14^N	[214]
EA-IRMS	Roasted coffee	67	Brazil	δ^13^C, δ^15^N, δ^18^O, and δ^2^H	[215]
GC-C-IRMS	Green and roasted coffee	320	Vietnam, Brazil, Cameroon, India, El Salvador, Ethiopia	δ^13^C	[216]
EA-IRMS	Green coffee	81	Ethiopia	δ^13^C, δ^15^N, and δ^18^O	[217]
Wine and related matrices					
GC-P-IRMS	Several wine varietals	110^+^	Europe	δ^18^O	[218]
GC-C-IRMS	Traditional and Moscatel Sparkling wine	36	Brazil	δ^13^C-CO_2_	[219]
GC-P-IRMS	Cabernet Sauvignon, Riesling, Pinot noir, Merlot, Cabernet Gernischet, Chardonnay, Longyan, Crystal and Rose honey	188	China	δ^18^O	[220]
SNIF-NMR-IRMS	Xynisteri, Maratheftiko, Cabernet Sauvignon, Shiraz	76	Cyprus	δ^2^H, δ^13^C, and δ^18^O	[221]
SNIF-NMR-IRMS	White (Fiano-Verdicchio) and red (Refosco-Nero) wines	16	Italy	δ^2^H, δ^13^C and δ^18^O	[222]
SNIF-NMR-IRMS	Balsamic vinegar must	27	Italy	δ^2^H and δ^13^C	[223]
Honey					
EA-IRMS	Raw and commercial	54	Australia, China, India, Indonesia, Iran, South Korea, China, Greece, Hungary, Macedonia, Romania, Serbia, New Zealand	δ^13^C	[192]
EA- and LC-IRMS (on organic acids)	Commercial	116	Japan, Spain, France, New Zealand, Italy, China, Hungary, Argentina, Bulgaria, Canada, Mexico, Romania, Taiwan, USA	δ^13^C	[224]
EA-IRMS	Commercial	17	Russia	δ^13^C	[225]
Coconut water					
EA-IRMS	Commercial/Industrialized	17	Brazil	δ^13^C	[211]

**Table 6:** Selected applications for vibrational spectroscopy used for juice and meat authenticity.

Matrix	Adulterant	Technique	Markers Used	Reference
Fruit juices, nectars, or pulps				
Concord Grape	Grape juice blends	FT-IR	Phenolic compound-rich fraction	[247]
Orange	Added sugar	FT-IR	Whole spectra	[248]
Passion fruit and guava	Water	NIR	Whole spectra	[249]
Orange	Added sugar	FT-IR	Fructose, glucose, and sucrose	[250]
Orange	Concentrate vs. fresh squeezed	ATR-FTIR	Whole spectra	[251]
Grape	Apple and cashew juice	MIR/ATR-0FTIR	Whole spectra	[70,71]
Grape, orange, peach, and passion fruit	Syrup, apple, cashew	MIR/ATR-FTIR	Whole spectra	[72]
Guava	Sugar and water	NIR and MIR	Whole spectra	[252]
Spirit drinks				
Distilled and aged ethanol	Counterfeit alcohol/ denaturants and additives	Raman	C-C stretch at 892, C-O stretch at 1059 and 1097, and CHx bend at 1460 cm^−1^	[253]
Natural drinks				
Coconut water	Sugar and water	Raman	Fructose, glucose, and sucrose at 627, 835, and 1123 cm^−1^	[254]
Meat tissue				
Bovine	NaCl, phosphates, carrageenan, maltodextrin	ATR-FTIR	Whole spectra	[255]
Bovine	Salts and carrageenan	Raman	Whole spectra	[256]
Salmon	Water	NIR	Whole spectra	[257]
Beef and mutton	Pork	FT-IR	Whole spectra	[258]

**Table 7:** Selected examples of OXITEST^®^ to assess food shelf life.

Country	Matrix Tested	Application	Induction Period (h) or Shelf Life and Temperature (°C, days)	Reference
Italy	Chia seeds	Differential analysis by country	13.01	[268]
Brazil	Rosemary leaves emulsion	Antioxidant capabilities	24.45	[269]
Italy	Bakery snack/Tarallini	Shelf life	20.90 (using extra virgin olive oil)	[270]
Kosovo	Cured and fresh meats	Effect of the addition of nitrates and chili peppers	15.11 (sausage with onions and peppers)	[271]
Italy	Tarallini	Enrichment with Tyrosyl oleate	25.28	[272]
China	Steam pork belly	Enrichment with pickled and dried mustard/Nutritional quality	Days	[273]
Thailand	Animal feed	Addition of tamarind polyphenols	5.42/5.43	[274]
Italy	Walnut paste	Enrichment with grape skin extract	13.85	[275]
Turkey	Sunflower oil	Effects of surfactants on emulsions	13.42	[276]
Russia	Oregano extract	Antioxidant capabilities	0.52	[277]
Italy	Sunflower oil	Enrichment with polyphenols from olive mill wastewater	17.03	[278]
China	God’s flower	Potential oil source	Days	[279]

**Table 8:** Examples of GC/MS techniques applied to the speciation of Hg.

Matrix	Mercury Species	Extraction Method	Chromatographic Conditions/Ions, m/z	Concentration Range, μg·kg^−1^ or μg·L^−1^	Reference
Cod, tuna, mackerel, and bonito	MeHg	Acetone, toluene, KBr, CuSO_4_, cysteine, HCl, NaBPh_4_, Na_2_SO_4_, PEG200	Inertcap 5MS/NP	265 to 294 (Validation data)	[295]
Tuna, shortfin squid, blue mussel, oyster, squid, tiger prawn, crown conch, hake, and salmon	MeHg and EtHg	MeOH and KOH, copper acetate, hexane, freeze-drying	TG-5MS/292, 294, 279 (Hg^2+^) and 308 306, 279	59.46 to 497.10 and 60.08 to 510.93	[296]
Water	Hg^2+^, MeHg, and EtHg	Sodium acetate, NaBPh_4_, and PDMS fiber SPME	Headspace, HP-5MS	1.2 × 10^−4^ to 5.0 × 10^−2^	[297]

**Table 9:** Examples of GC/MS techniques applied to the flavor profile of cocoa.

Country	Matrix Analyzed	Pyrazine Compounds	Extraction Method	Chromatographic Conditions	Concentration Range	Reference
Papa New Guinea, Ivory Coast, Indonesia, Ghana, Cameroon	Cocoa liquors	2,3-/2,5-/2,6-diMePy, EtPy, triMePy, 2-Et-3,5-diMePy, 3-Et-2,5-diMePy, tetraMePy, 3,5-diEt-2-MePy	Purge and Trap concentrator	DB-WAX and DB-5MS	11.19 (triMePy/Indonesia)-532.37 (tetraMePy/Papa New Guinea) (ng/g)	[303]
Brazil	Cocoa beans and chocolates	2,3,5,6-tetraMePy, 2,3,5-triMePy	Maceration liquid nitrogen, SPME (DVB/CAR/PDMS)	Headspace, OV Carbonwax 20M	Qualitative	[304]
Ivory Coast	Cocoa powder	2,3,5,6-tetraMePy, 2-Et-3-Py, 2,5-diMePy, 2,3,5-triMePy	Water, Likens–Nickerson, hexane, Na_2_SO_4_	HP-5 MS	0.23–2.69 (g/100 g relative area)	[305]
USA/Ghana	Dark chocolate	2,6-diMePy, tetraMePy, 2,3,5-trimethyl 6-ethyl pyrazine	10 min 60 °C, SPME (DVB/CAR/PDMS)	Headspace, DB-WAXUI Ultra Inert	Qualitative	[306]
Indonesia	Dark and milk chocolate	2,3-/2,5-/2,6-diMePy, triMePy, tetraMePy, 2-Et-5-MePy, MePy	30 min 55 °C, SPME	Not indicated	0.13 to 1.21 (g/100 g relative area)	[307]

**Table 10:** Examples of LC/MS^n^ techniques applied to allergen detection in foods.

Matrix Tested, Allergens Analyzed or Peptide Sequence	Extraction Method/Digestion	Chromatographic Conditions/LC System	Reference
Single allergen approaches			
Soybena grains, soybean and bovine milk, soy flour, fruit and vegetable juices/Gly m 4	Tris-HCl, shaking 1 h, centrifugation, trypsin digestion, SPE clean-up (OASIS^®^ MCX), centrifugation Nanosep^®^ membrane	3200QTRAP, ESI^+^, AdvanceBio Peptide Map, 2.1 × 150 mm, 2.7 μm	[377]
*P. muelleri* (LTNAVNEIEKR), *P. borealis* (SFLVWVNEEDQLR), *P. monodon* (AVFDQLKEK/VSSTLSSLEGELK/ TFLVWVNEEDHLR/LEEVAGKYNLQVR), *L. vannamei* (VSSTLSSLEGELK/TFLVWVNEEDHLR/ LEEVAGKYNLQVR), *F. merguiensis* (ALFDQLKDKK/TFLVWVNEEDHLR/LEEVAGKYNLQVR), *F. indicus* (TFLVWVNEEDQLR/LEEVAGKYNLQVR), *F. notialis* (VSSTLSSLEGELK/TFLVWVNEEDHLR).	Dispersion in H_2_O, centrifugation, sonication, desalted trypsin digests	nano-electrospray ionization (ESI)-ion trap (IT), BioBasic-18 RP 0.18 × 150 mm, SMIM,	[378]
Fish. Parvalbumin (e.g., LFLQTFSAGAR)	Tris-HCl dispersion of muscle, centrifugation, trypsin digestion using high-intensity ultra sound	LTQ LIT (linear ion trap) ESI^+^, reverse phase C_18_ gradient ACN and H_2_O, SMIM, e.g., 709.36 m/z	[379]
Beer. Gluten (hordein, glutenin, γ-secalin, γ-prolamin, and γ-gliadin)	Degassification, centrifugation, dithiotretol reduction, iodoacetamide, trypsin digestion	nanoESI^+^ TripleTOF^TM^, Zorbax300SB-C18 150 mm × 75 μm	[380]
Cereals. Barley/Gluten (VFLQQQCSPVR)	2-propanol and dithiotreitol 60 °C, centrifugation, iodoacetamide alkylation (prevent re-oxidation of cysteine residues), trypsin digestion	6500 QTRAP, MRM (see reference immediately above)	[381]
Cookies. Casein α_S1_ (FFVAPFPEVFGK)	NH_4_HCO_3_/(NH_4_)_2_CO_3_, SDS, centrifugation, trypsin	3D ion trap ESI^+^, ACE C18-300 Å 250 × 1 mm,	[382]
Multiple allergen approaches			
Multiple commercial products. e.g., nutter bar, protein bar, nut crisps. Roasted and native peanut and tree nuts (e.g., AHVQVVDSNGDR/SFNLDEGHALR/GTGNLELVAVR/TANDLNLLILR).	Tris-HCl, 2 h 50 °C, centrifugation trypsin digestion.	6530 q-TOF *Protein analysis* Poroshell 300 2.1 × 75 mm 2.7 μm, 300–2800 m/z *Peptide analysis* ESI^+^, Poroshell 120 2.1 × 50 mm 2.7 μm,	[383]
Cookies. Ovalbumin (EVVGSAEAGVDAASVSEEFR/GGLEPINFQTAADQAR/LTEWTSSNVMEER/YPILPEYLQCVK) for eggs, β-lactoglobulin (TPEVDDEALEK), α_s_-casein (YLGYLEQLLR/FFVAPFPEVFGK) for milk, β-conglycinin-α-chain (ESYFVDAQPK/TISSEDKPFNLR) for soy	Tris-HCl, G25 Sphadex, Ultrasound, Trypsin digestion and Rapidigest^TM^ as surfactant/denaturing agent	ESI^+^ Linear IonTrap Dual Velos Pro^TM^, Acclaim^TM^ PepMap 1 mm × 15 cm × 3 μm, at 0.06 mL·min^−1^ ACN and H_2_O, SRM 1004.98/844.42/799.36/761.90; 623.30; 634.36/692.87; 592.23/703.87 m/z	[384]
Cookies, cake. α-La (VGINYWLAHK), β-Lg (LIVTQTMK/TPEVDDEALEK), α_s1_-CN (HQGLPQEVINENLLR/YLGYLEQLLR)	Tris-HCl, centrifugal filter, SDS-PAGE, acetylation iodoacetamide, Trypsin digestion	BEH300 C_18_ 2.1 × 100 mm, 1.7 μm, Q-TOF ESI^+^ (Cooroborated by MALDI-TOF-TOF), 601.1/931.5; 601.1/654.4; 623.8/199.2; 623.8/1048.2; 634.6/249.2, 634.6/991.3 m/z, MRM	[385]
Cookies, bread, cookie dough, salada dressing, white wine, infant formula, dark and milk chocolate, ice cream, breakfast cereal. Egg white, egg yolk, milk, peanut, hazelnut, pine nut, Brazilian nut, cashew, pecan, soy, almond.	Defat in hexane, Trizma base for deproteinization, octyl β-D-glucopyranoside, tris(2-carboxyethyl)phosphine hydrochloride, *S*-methyl methanethiosulfonate, CaCl_2_, NH_4_HCO_3_, trypsin digestion	Screening. UFLC_XR_, MRM, ESI^+^ Quantification. ExionLC AD, QTRAP 6500, IonDrive Turbo V Ion Source, MRM	[386]
Bakery products and chocolates. Peanut Pistachio, Hazelnut, Almond, Cashew Walnut	Dispersion, trypsin digestion, C_18_ SPE	QTRAP 6500, IonDrive^TM^ Tubo V ESI^+^, Phenomenex Kinetex, 2.6 μm, C18, 100 × 2.1 mm at 0.3 mL·min^−1^ ACN and H_2_O, MRM	[387]

**Table 11:** Examples of LC/MS^n^ techniques to assess vanillin fraud.

Food Product	Country	Target Compounds	LC System	Chromatographic Conditions	Concentrations Found, mg·mL^−1^ or mg·g^−1^	Reference
Vanilla extracts	USA, Mexico, Peru, Dominican Republic	Coumarin Vanillin 3′,4′-(methylenedioxy)acetophenone (as IS) Ethyl vanillin	LC-UV-MS, ESI^+^ SIM, 147, 153, 165, 167 m/z, λ = 254 nm	Luna 5 μm ODS C_18_ 250 × 2.0 mm, 0.25 mL·min^−1^, isocratic elution ACN and H_2_O	Vanillin_authenthic_ = 1.12–1.61 Vanillin_artifical_ = 1.95–8.59 Ethyl vanillin_artifical_ = 0.33–0.65	[392]
*V. planifolia* J. W. Moore and *V. tahitensis* G. Jackson cured beans	Mexico, Reunion Island India, Costa Rica, Madagascar, Papa New Guinea, French Polynesia	*p*-hydroxybenzyl alcohol, protocatechuic acid, vanillyl alcohol, protocatechualdehyde, *p*-hydroxybenzoic acid, vanillic acid, *p*-hydroxybenzaldehyde, isovanillin, vanillin, anisyl alcohol, methyl *p*-hydroxybenzoate, anisic acid, anisaldehyde, methyl anisate	LC-UV-MS, ESI^+^ SIM, 147, 153, 165, 167 m/z, λ = 260 nm	λ = 260 and 280 nm, Superspher 100 RP C18, 250 × 4 mm, 4μm	*V. planifolia* 1.7–3.6 dwb; *V. tahitensis* 1.0–2.0 dwb	[393]
Infant Formula	China	Coumarin Vanillin Ethyl vanillin vanillin-^13^C_6_ and coumarin-D_4_	LC-QqLIT-MS 153.0, 167.0, 147.0 m/z	Waters XSelect HSS T3 150 × 2.1 mm and 3.5–μm, gradient ACN and H_2_O	Vanillin = 0.0023 to 0.71	[394]
